# Prognosis stratification of cancer patients treated with immune checkpoint inhibitors through lung immune prognostic index: a meta-analysis and systematic review

**DOI:** 10.1186/s12885-024-12271-0

**Published:** 2024-04-25

**Authors:** Yusheng Guo, Yao Pan, Jiayu Wan, Bingxin Gong, Yi Li, Xuefeng Kan, Chuansheng Zheng

**Affiliations:** 1 Department of Radiology, Union Hospital, Tongji Medical College, Huazhong University of Science and Technology, 430022 Wuhan, China; 2 Hubei Key Laboratory of Molecular Imaging, 430022 Wuhan, China; 3 Department of Obstetrics and Gynecology, The Second Affiliated Hospital of Fujian Medical University, 362000 Quanzhou, China

**Keywords:** Lung immune prognostic index, Immune checkpoint inhibitors, Prognosis stratification

## Abstract

**Background:**

Although numerous studies have reported the prognostic value of the lung immune prognostic index (LIPI) in non-small cell lung cancer (NSCLC) patients treated with immune checkpoint inhibitors (ICIs), the prognostic value of the LIPI in a pancancer setting remains unclear.

**Methods:**

A comprehensive search was conducted until July 2023 across the PubMed, Embase, Web of Science, and Cochrane Library databases to identify relevant studies evaluating the prognostic value of the LIPI in cancer patients treated with ICIs. The outcomes were overall survival (OS), progression-free survival (PFS), objective response rate (ORR), and disease control rate (DCR). We described and compared the pooled outcomes by stratifying the patients based on different groupings of LIPI (good vs. intermediate [0 vs. 1], good vs. poor [0 vs. 2], and good vs. intermediate / poor [0 vs. 1 + 2]).

**Results:**

A total of 9959 patients in 35 studies were included. A higher score of LIPI was associated with impaired OS. The pooled HRs were 1.69 (95% CI: 1.55–1.85, *p* < 0.001; 0 vs. 1), 3.03 (95% CI: 2.53–3.63, *p* < 0.001; 0 vs. 2), and 2.38 (95% CI: 1.97–2.88, *p* < 0.001; 0 vs. 1 + 2). A higher LIPI score was associated with shorter PFS. The pooled HRs were 1.41 (95% CI: 1.31–1.52, *p* < 0.001; 0 vs. 1), 2.23 (95% CI: 1.87–2.66, *p* < 0.001; 0 vs. 2), and 1.65 (95% CI: 1.46–1.86, *p* < 0.001; 0 vs. 1 + 2). Similarly, a higher LIPI score was associated with a lower ORR. The pooled ORs were 0.63 (95% CI: 0.54–0.75, *p* < 0.001; 0 vs. 1) and 0.38 (95% CI: 0.29–0.50, *p* < 0.001; 0 vs. 2). A higher LIPI score was associated with a lower DCR. The pooled ORs were 0.47 (95% CI: 0.35–0.61, *p* < 0.001; 0 vs. 1) and 0.19 (95% CI: 0.12–0.30, *p* < 0.001; 0 vs. 2).

**Conclusion:**

In patients with NSCLC or other solid tumours, the lung immune prognostic index could robustly stratify the clinical outcomes into three groups among the patients who receive ICIs. LIPI is a low-cost, simple, accessible, and accurate prognostic tool in a pancancer setting and it may contribute to the evaluation of risk stratification in patients treated with ICIs.

**Supplementary Information:**

The online version contains supplementary material available at 10.1186/s12885-024-12271-0.

## Introduction

Over the past decade, the utilization of immunotherapy has substantially transformed the therapeutic domain of numerous solid tumours [1]. Immune checkpoint inhibitors (ICIs) that specifically target programmed cell death-1/programmed death ligand-1 (PD-1/PD-L1) and T-lymphocyte-associated protein 4 (CTLA-4) are particularly noteworthy [2]. Immune checkpoint inhibitors (ICIs) have demonstrated notable efficacy in enhancing overall survival rates in various cancer types, such as non-small cell lung cancer (NSCLC), melanoma, renal cell carcinoma, and hepatocellular carcinoma (HCC) [3]. However, despite considerable achievements, considerable variability in treatment response and survival outcomes is observed among patients undergoing ICI therapy [4], therefore, it is imperative to ascertain suitable biomarkers capable of identifying patients who may not derive substantial benefits from ICI treatment to avert the administration of futile, costly, and potentially harmful interventions [5].

Despite the increasing number of studies investigating prognostic biomarkers in ICI therapy, such as PD-L1 expression, tumour mutational burden (TMB), or mismatch repair deficiency (dMMR) [6–8], there is a notable absence of a universally applicable clinical tool. In order to validate these biomarkers, next-generation sequencing (NGS) or immunohistochemical analysis is required [9]. However, the biopsy site and specimen status can influence the results. Consequently, there is a need to identify readily accessible biomarkers that are suitable for accurately predicting the efficacy of ICIs treatment across different tumor types in various clinical settings. The immune status of the tumour microenvironment has been shown to be a key indicator of antitumour immune responses [10]. Systemic chronic inflammation can dysregulate immune homeostasis and suppress the adaptive antitumour immune response [11]. As a representative mediator of systemic inflammation, cancer cell-regulated neutrophils can inhibit the anti-tumor function of T cells, which may hinder the efficacy of immunotherapy [12]. Prior studies have indicated the importance of the baseline neutrophil-to-lymphocyte ratio (NLR) and the baseline lactate dehydrogenase (LDH) level in prognostically assessing the outcomes in different types of cancer [13–15]. LDH is a biomarker of metabolism and proliferation. Serum LDH levels reflect the overall burden of the tumour and reflect its invasiveness [16]. Consequently, to strengthen the prognostic power of these two indexes, the lung immune prognostic index (LIPI), which combines the derived NLR (dNLR) and LDH, was proposed as a means of identifying NSCLC patient subgroups with differential tumour responses and survival outcomes after ICI treatment [17, 18]. This inexpensive and readily available index included a pretreatment dNLR greater than 3 and an LDH level higher than the upper limit of normal, stratifying patients into “poor”, “intermediate” and “good” prognostic groups [18].

Recently published studies have highlighted the potential prognostic value of LIPI in solid cancer patients undergoing ICI treatment beyond NSCLC [17, 19–33]. Although a considerable number of studies have investigated the association between LIPI and the prognosis of patients treated with ICIs, a comprehensive review on this topic is currently lacking. Hence, we conducted this meta-analysis and systematic review to comprehensively summarize the prognostic importance of the LIPI in patients receiving ICIs in a pancancer setting.

## Methods

### Search strategy

This meta-analysis and systematic review adhered to the Preferred Reporting Items for Systematic Reviews and Meta-Analyses guidelines [34] and the protocol for the analysis was registered prospectively in PROSPERO (CRD42023441536). A comprehensive search of multiple databases including PubMed, Web of Science, the Cochrane Library, and Embase, was conducted to identify relevant studies published until July 2023. The search utilized specific keywords such as “lung immune prognostic index”, “LIPI”, “cancer”, “solid tumour”, “tumour”, “immunotherapy”, “ICI”, and “immune checkpoint inhibitor”. Furthermore, a manual scan of the references of the included studies was conducted to identify any potentially overlooked studies.

### Study selection

The preliminary literature review was conducted by two independent authors (Yusheng Guo and Yao Pan) who identified relevant studies by reading titles and abstracts in various databases. To be considered eligible for inclusion, studies had to meet the following criteria: (1) were published in English, (2) evaluated the prognostic value of LIPI in cancer patients treated with ICIs, and (3) reported outcomes such as overall survival (OS), progression-free survival (PFS), objective response rate (ORR), disease control rate (DCR), or immune-related adverse events (irAEs).

### Data extraction

The data extracted from the included studies encompassed various variables, including the year of publication, name of the first author, region, type of ICIs, type of tumour, outcomes, number of enrolled patients, and the ratio of males to females. The assessment of each study was independently conducted by two authors using the Newcastle-Ottawa scale (NOS), with studies scoring an NOS score ≥ 6 classified as high-quality studies. In the event of any disagreements, a resolution was achieved through discussion or consensus with a third author (Xuefeng Kan or Chuansheng Zheng). In instances where multiple publications reported overlapping data, priority was given to the study with the largest sample size or the study with more comprehensive information on LIPI. The primary endpoint of this study was OS, defined as the time from the initiation of treatment to death. The secondary endpoints were as follows:1) PFS, defined as the time from the initiation of treatment to progressive disease (PD) or death; 2) ORR, defined as the proportion of patients with complete response (CR) or partial response (PR); and 3) DCR, defined as the proportion of patients with CR, PR or stable disease (SD). The ancillary endpoint was irAEs.

### Statistical analyses

The statistical analysis was performed utilizing R software (version 4.1.0). Prior to conducting a meta-analysis, heterogeneity was assessed through the implementation of a chi-square test and the I^2^ metric. The I^2^ value serves as an indicator of the proportion of variability across the pooled estimates that can be attributed to statistical heterogeneity. Studies with an I^2^ value exceeding 35% were deemed to possess substantial heterogeneity. In instances of high heterogeneity, a random effects model was employed, while a fixed effects model was utilized in cases of low heterogeneity. Subsequently, the forest maps were created, followed by a comprehensive description and discussion of the HRs or ORs along with their corresponding 95% confidence intervals (CIs). Potential sources of heterogeneity were identified utilizing Baujat plots, and sensitivity analyses were subsequently performed by excluding studies one by one. Subgroup analyses of OS and PFS were performed based on patient characteristics. Publication bias was evaluated using funnel plots, Egger’s test, and Begg’s test. Every time the meta-analysis was conducted with a fixed effect model or a random effect model, a publication bias test was carried out. The results of Egger’s test and Begg’s test were presented in the table. In cases where publication bias was identified, the trim-and-fill method was employed to generate a model that accounted for such bias. A significance level of *p* < 0.05 was deemed statistically significant in all the statistical analyses.

## Results

**Fig. 1:**
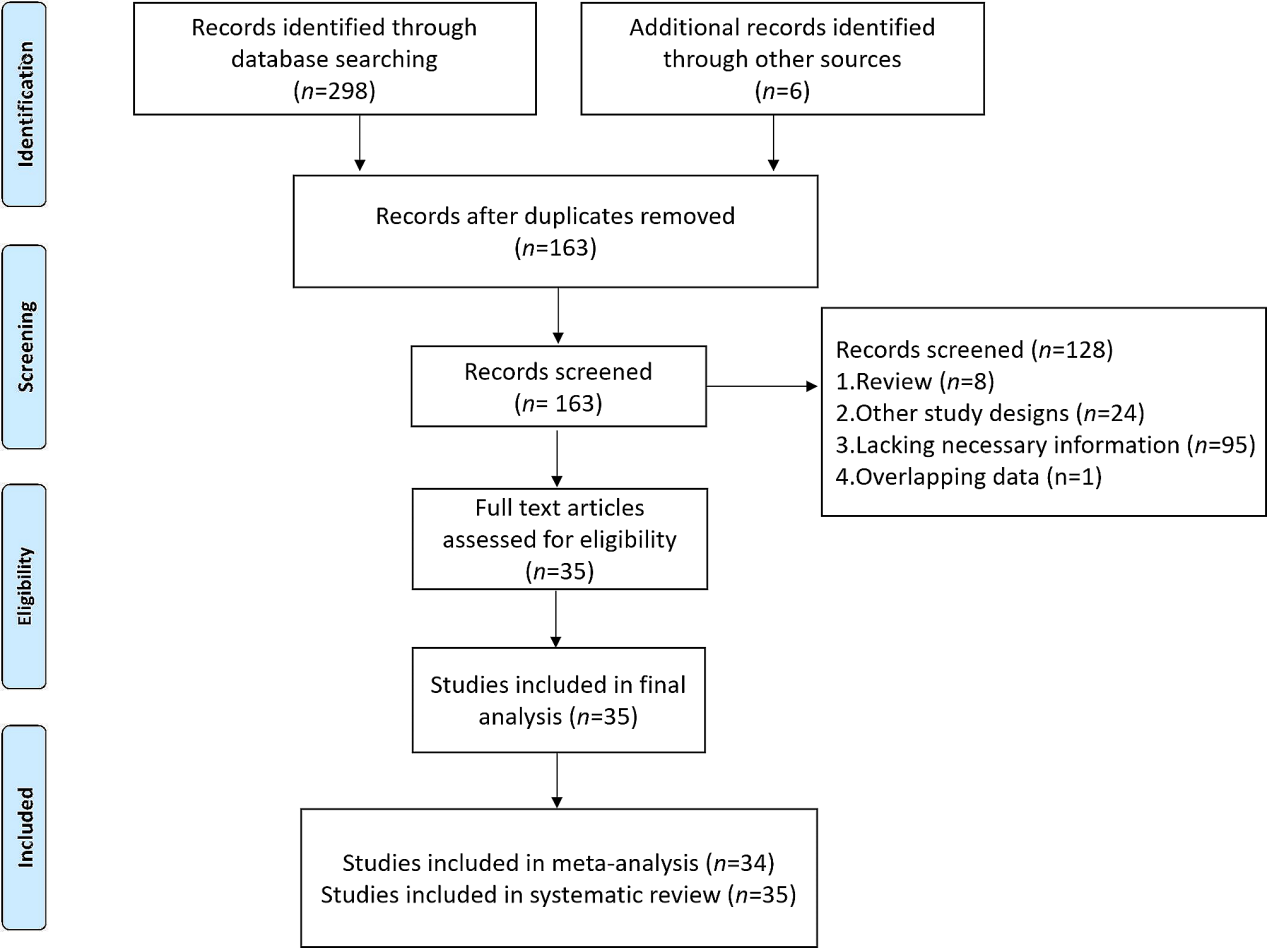
Flow diagram of study selection for inclusion in this meta-analysis and systematic review

After conducting a thorough screening of the databases, a total of 163 nonduplicated studies were identified. Subsequently, 128 studies were excluded based on predetermined criteria, leaving 35 studies for further evaluation through abstract review in accordance with the inclusion criteria [17–33, 35–52]. After a thorough examination of the full text, a total of 35 studies were included in this meta-analysis and systematic review (Fig. 1), and the study reported by Sonehara et al. [48] addressing irAEs was included into systematic review. Given that two studies by Hopkins et al. [53] and Sorich et al. [36] included duplicate patients, the study by Sorich et al., which included more comprehensive information on LIPI was included. Notley, Parent et al. [21], Mountzios et al. [42], Hopkins et al. [41], Uehara et al. [49], and Wang et al. [38] reported two immunotherapy cohorts. Therefore, forty-one cohorts were included in this study (Table 1).

### Characteristics of the included studies

**Fig. 2:**
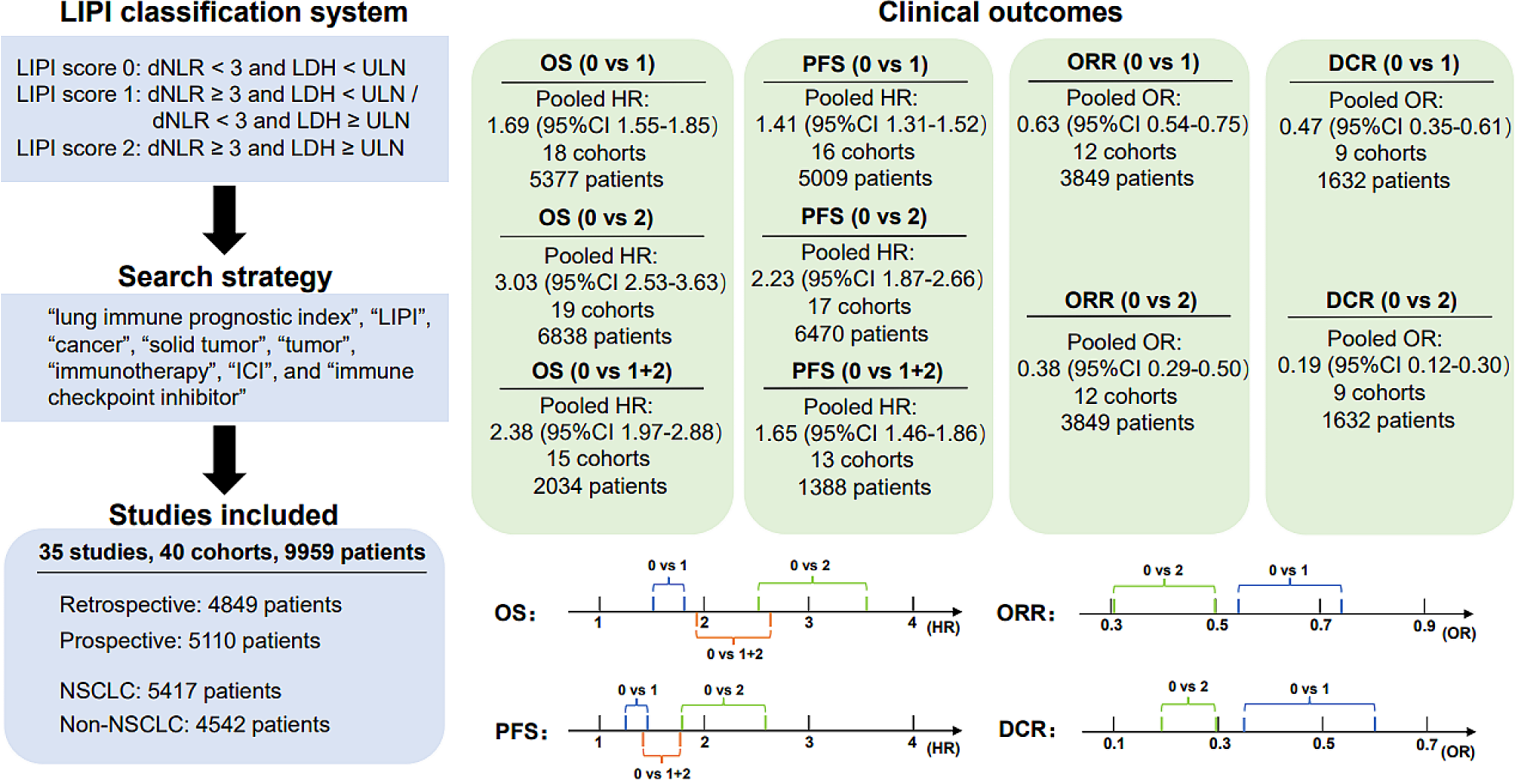
Overview of this study

A total of 35 studies (40 cohorts) involving 9959 cancer patients were included in the meta-analysis and systematic review [17–33, 35–52]. Thirty cohorts had a retrospective study design, and ten cohorts (9 studies) were prospectively designed or from prospective trials [17, 21, 22, 32, 36, 37, 40, 41, 51]. All the studies included in the analysis obtained moderately high scores on quality assessments conducted using the Newcastle-Ottawa Scale. Among the 35 studies that were included, twenty-nine focused on specific types of cancer, with non-small cell lung cancer (NSCLC) being the most commonly reported tumour [18, 32, 35–39, 41–46, 48–52]. Six studies addressed two or more types of tumors [17, 22, 23, 30, 32, 33]. The ICIs used in the aforementioned studies included PD-1 antibodies (nivolumab and pembrolizumab are commonly used), PD-L1 antibodies (atezolizumab is commonly used), CTLA-4 antibody (ipilimumab), and combination therapies including immunotherapy. Additionally, the LIPI classification system was employed to categorize the population into three groups, namely, good (0), intermediate (1), and poor (2). Consequently, we proceeded to describe and compare the clinical outcomes by stratifying the patients based on different groupings of LIPI (0 vs. 1, 0 vs. 2, and 0 vs. 1 + 2) to accurately assess the impact of each upgrade on patient clinical outcomes. The main process and results of this study are shown in Fig. 2.

**Table 1:** Characteristics of the included studies

Year	Author	Region	Treatment	Tumour	Number of patients	Outcomes	Sex (male/fem ale)	Newcastle-Ottawa scale	Study design*
2023	Tao Sun	China	camrelizumab	HCC	224	OS; PFS; ORR; DCR	182/42	7	R
2023	Magdalena Knetki-Wróblewska	Poland	nivolumab or atezolizumab	advanced NSCLC	260	OS; PFS	149/111	7	R
2023	Jingyuan Xie	China	anti-PD-1 or anti-PD-L1	extensive-stage small cell lung cancer	116	OS; PFS	106/10	7	R
2023	Pauline Parent	France, Spain, Belgium, and other	anti-PD1 and anti‐PDL1 (ICI cohort)	advanced or metastatic urinary tract carcinoma	137	OS; PFS	112/25	7	R
2023	Pauline Parent	France, Spain, Belgium, and other	atezolizumab (SAUL cohort)	advanced or metastatic urinary tract carcinoma	541	OS; PFS; ORR; DCR	408/133	8	P
2023	Omer Diker	Nicosia	/	NSCLC	150	OS; PFS; ORR	131/19	6	R
2023	Javier García-Corbacho	Spain	/	advanced solid tumours	146	OS; PFS; ORR	/	7	P
2023	Andrea De Giglio	Italy	/	advanced NSCLC	183	OS	114/69	7	R
2023	Monica Pierro	France	pembrolizumab, nivolumab, atezolizumab, and, avelumab	solid tumours	191	OS; PFS; ORR; irAEs	117/74	7	R
2023	Yusuke Inoue	Japan	atezolizumab	NSCLC	69	OS; PFS	/	7	P
2022	Miaomiao Gou	China	nivolumab, pembrolizumab, sintilimab, and, toripalimab	metastatic gastric cancer	186	OS	132/54	7	R
2022	Kei Sonehara	Japan	nivolumab, pembrolizumab, and, atezolizumab	NSCLC	113	irAEs	91/22	7	R
2022	Jia Chen	China	pembrolizumab, nivolumab, sintilimab, and, toripalimab.	advanced NSCLC	85	OS; PFS	62/23	6	R
2022	Ana Ortega-Franco	UK	pembrolizumab	NSCLC	113	OS; PFS; ORR; DCR	67/46	7	R
2022	Jean-Baptiste Assié	French	nivolumab	malignant pleural mesothelioma	109	OS	74/35	7	R
2022	Shiyun Chen	China	nivolumab, pembrolizumab, and, sintilimab	pancreatic carcinoma	98	OS; PFS	70/28	7	R
2022	Yuji Uehara	Japan	ICI-monotherapy	NSCLC	80	PFS	62/18	6	R
2022	Yuji Uehara	Japan	ICI-chemotherapy	NSCLC	80	PFS	62/18	6	R
2022	Yuting Pan	China	nivolumab, toripalimab, sintilimab, and, pembrolizumab	advanced bile tract carcinoma	110	OS; PFS	67/43	6	R
2021	G Mountzios	Greece and Germany	ICI-monotherapy	advanced NSCLC	206	OS; PFS	125/81	7	R
2021	G Mountzios	Greece and Germany	ICI-chemotherapy	advanced NSCLC	206	OS; PFS	125/81	7	R
2021	Edouard Auclin	France and Spain	atezolizumab, avelumab, nivolumab, ipilimumab, and, pembrolizumab	mismatch repair deficient tumours	151	OS; PFS	62/89	7	R
2021	Wael Abdullah Sultan Ali	China	pembrolizumab, nivolumab, camrelizumab, and, atezolizumab	NSCLC	73	OS; PFS	51/22	6	R
2021	Jiamin Sheng	China	pembrolizumab, nivolumab, atezolizumab, and, sintilimab	NSCLC	41	OS; PFS	28/13	6	R
2021	Baicun Hou	China	/	advanced gastric cancer	120	OS; PFS; ORR; DCR	80/40	7	R
2021	Antonello Veccia	Italy	pembrolizumab	NSCLC	117	ORR; DCR	78/39	6	R
2021	Heidi A I Grosjean	Canada	pembrolizumab	PD-L1 Positive NSCLC	327	OS	157/170	7	P
2021	Ashley M Hopkins	Australia	atezolizumab (ABCP)	advanced NSCLC	400	OS; PFS	240/160	7	P
2021	Ashley M Hopkins	Australia	atezolizumab (ABP)	advanced NSCLC	402	OS; PFS	241/161	7	P
2021	Lingling Li	China	anti-PD-1 or anti-PD-L1	advanced small cell lung cancer	100	OS; PFS	88/12	6	R
2020	G Mazzaschi	Italy	pembrolizumab, nivolumab, and, atezolizumab	NSCLC	109	OS	73/36	7	P
2020	Ghassan Al Darazi	France	/	solid tumour	259	OS	169/90	7	P
2020	Wenxian Wang	China	ICI-monotherapy	NSCLC	216	OS; PFS	179/39	6	R
2020	Wenxian Wang	China	ICIs combined with chemotherapy	NSCLC	114	OS; PFS	98/16	6	R
2020	Shixue Chen	China	nivolumab, pembrolizumab, and, other	HCC	108	OS; PFS	90/18	7	R
2019	Juan Ruiz-Bañobre	Spain	nivolumab	advanced NSCLC	153	OS; PFS; ORR	119/34	7	R
2019	Daniel E Meyers	Canada	nivolumab, pembrolizumab, and, ipilimumab/nivolumab	NSCLC, RCC, and melanoma	578	OS; PFS; ORR	/	7	R
2019	Michael J Sorich	Australia	atezolizumab	advanced NSCLC	1489	OS; PFS	/	7	P
2019	Dickran Kazandjian	USA	/	/	1368	OS; PFS	/	7	P
2018	Laura Mezquita	Europe	nivolumab, pembrolizumab, atezolizumab, durvalumab, and, durvalumab-ipilimumab	NSCLC	431	ORR; DCR	301/130	7	R

Note. NSCLC: non-small cell lung cancer; RCC: renal cell carcinoma; HCC: hepatocellular carcinoma; OS: overall survival; PFS: progression-free survival; ORR: objective response rate; PD-1: programmed death-1; PD-L1: programmed death ligand-1; irAEs: immune-related adverse events; ^*^P: prospective; R: retrospective

### Overall survival and LIPI

**Fig. 3:**
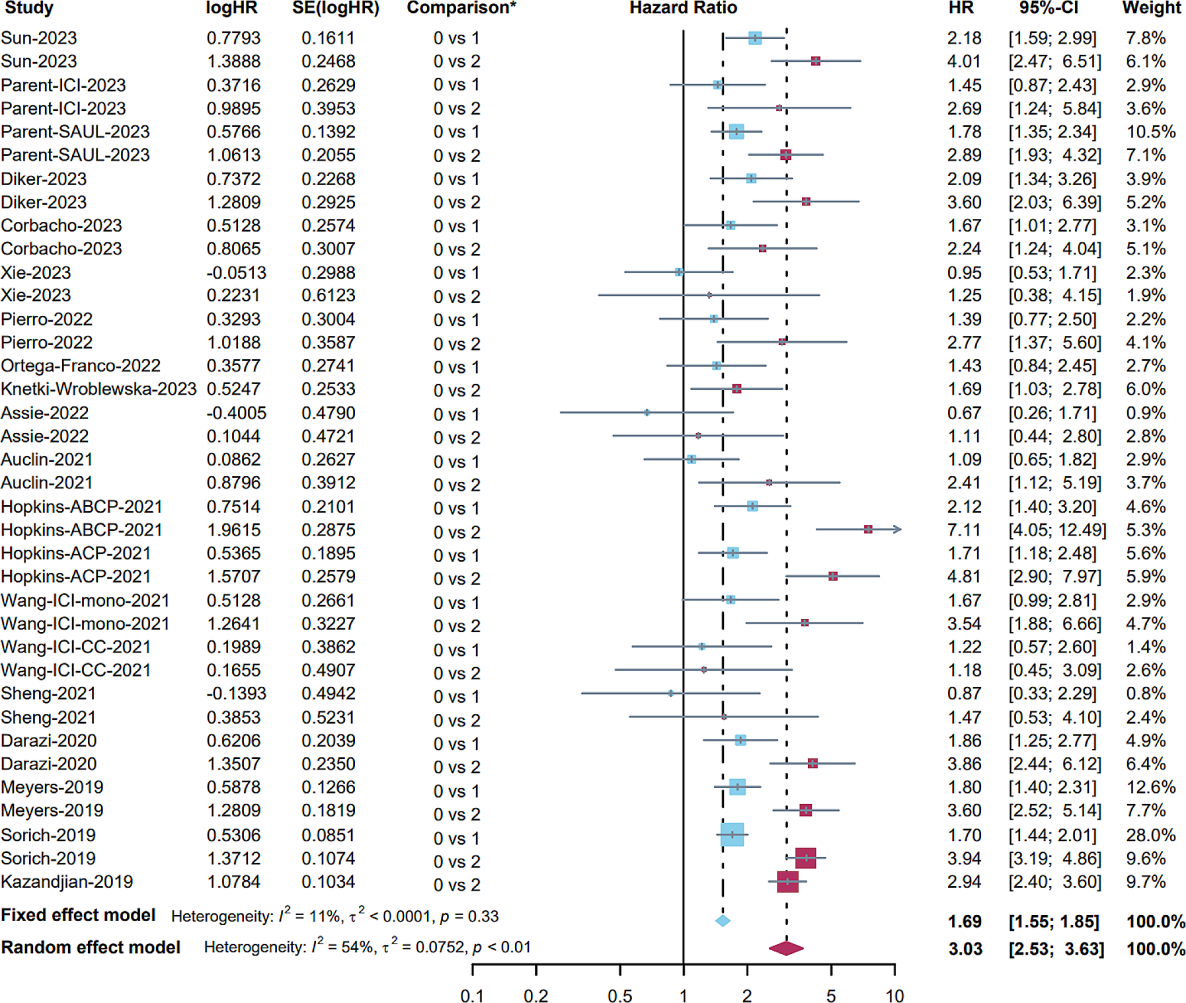
Forest plot for the association between LIPI 1 and OS (blue); the association between LIPI 2 and OS (red)

Among the 35 publications selected for the meta-analysis, a total of 31 studies (comprising 35 cohorts) contributed data for the primary endpoint, OS. To intuitively observe the associations between different levels of LIPI (0 vs. 1 and 0 vs. 2) and OS, we combined these two meta-analyses in the same forest plot (Fig. 3), and the results are shown in different colours (0 vs. 1: blue and 0 vs. 2: red). Fifteen studies (18 cohorts) involving 5377 patients reported an association between OS and LIPI (0 vs. 1) [19–23, 27, 30, 32, 33, 36, 38, 41, 43, 47, 50]. Considering the low heterogeneity (I^2^ = 11%), a fixed effects model was used for analysis. The results indicated that the pooled HR was 1.69 (95% CI: 1.55–1.85, *p* < 0.001; Fig. 3), suggesting the prognostic role of LIPI in patients who received immunotherapy. Sixteen studies (19 cohorts) involving 6838 patients reported an association between OS and LIPI (0 vs. 2) [17, 19–23, 27, 30, 32, 33, 36, 38, 41, 43, 50, 52]. There was high heterogeneity (I^2^ = 54%) in these studies, and a random effects model indicated that LIPI 2 was associated with shorter survival (pooled HR: 3.03, 95% CI: 2.53–3.63, *p* < 0.001; Fig. 3). Notably, LIPI 2 indeed represented more impaired survival than LIPI 1. The Baujat plot showed that the studies by Hopkins et al. [41] and Sorich et al. [36] contributed the maximum heterogeneity and influence to the overall result, respectively (Figure S1). Leave-one-out sensitivity analyses were conducted to assess the robustness of the results of meta-analyses by excluding the included studies one by one (Figure S2).

Fourteen studies (15 cohorts) investigated the association between OS and LIPI (0 vs. 1 + 2) [24–26, 28, 29, 31, 35, 37, 39, 40, 42, 45, 46, 51]. Given the high heterogeneity (I^2^ = 46%), a random effects model was used for analysis, and the results showed that LIPI 1 or 2 was associated with poor survival outcomes (pooled HR: 2.38, 95% CI: 1.97–2.88, *p* < 0.001; Figure S3). The Baujat plot showed that the study by Gou et al. [25] contributed the maximum heterogeneity and influence to the overall results (Figure S4), and the leave-one-out sensitivity analyses showed stable results (Figure S5).

Subgroup analyses of OS (0 vs. 1 and 0 vs. 2) were conducted according to retrospective or prospective and tumour type. The results indicated that all subgroups provided similar results (Table S1). Notably, the subgroup analysis of the prospective studies provided similar results to the results of retrospective studies (pooled HR for 0 vs. 1 in retrospective studies: 1.64, 95% CI: 1.45–1.85, *p* < 0.001; pooled HR for 0 vs. 1 in prospective studies: 1.75, 95% CI: 1.54-2.00, *p* < 0.001; pooled HR for 0 vs. 2 in retrospective studies: 2.60, 95% CI: 2.08–3.25, *p* < 0.001; pooled HR for 0 vs. 2 in prospective studies: 3.79, 95% CI: 2.92–4.91, *p* < 0.001). In addition, the NSCLC subgroup provided similar results to non-NSCLC subgroup (pooled HR for 0 vs. 1 in NSCLC studies: 1.71, 95% CI: 1.51–1.94, *p* < 0.001; pooled HR for 0 vs. 1 in non-NSCLC studies: 1.62, 95% CI: 1.38–1.89, *p* < 0.001; pooled HR for 0 vs. 2 in NSCLC studies: 3.15, 95% CI: 2.27–4.38, *p* < 0.001; pooled HR for 0 vs. 2 in non-NSCLC studies: 3.03, 95% CI: 2.55–3.60, *p* < 0.001).

### Progression-free survival and LIPI

Twenty-six studies (31 cohorts) investigated the association between secondary point (PFS) and LIPI. Similarly, we combined these two meta-analyses (0 vs. 1 and 0 vs. 2) in the same forest plot (Fig. 3), and the results are shown in different colours (0 vs. 1: blue and 0 vs. 2: red). Thirteen studies (16 cohorts) involving 5009 patients reported an association between PFS and LIPI (0 vs. 1) [19–23, 30, 33, 36, 38, 41, 43, 47, 50]. The fixed effects model indicated that LIPI 1 was associated with shorter survival (pooled HR: 1.41, 95% CI: 1.31–1.52, *p* < 0.001, I^2^ = 0%, Fig. 3).

**Fig. 4:**
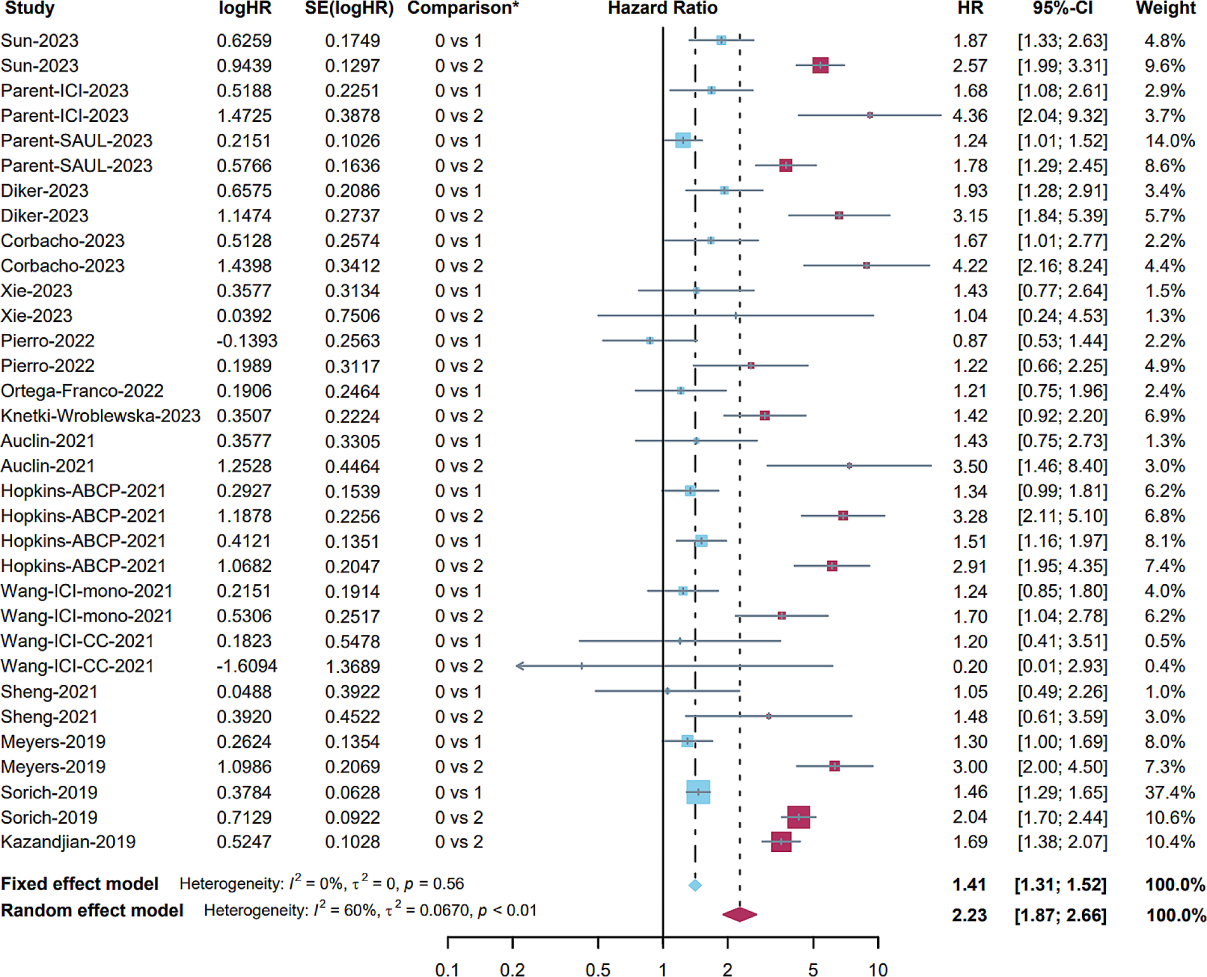
Forest plot for the association between LIPI 1 and PFS (blue); the association between LIPI 2 and PFS (red)

Fourteen studies (17 cohorts) involving 6470 patients reported an association between PFS and LIPI (0 vs. 2) [17, 19–23, 30, 33, 36, 38, 41, 43, 50, 52]. Similarly, the random effects model indicated that LIPI 2 was associated with shorter survival (pooled HR: 2.23, 95% CI: 1.87–2.66, *p* < 0.001, I^2^ = 60%, Fig. 4). Despite the heterogeneity, the leave-one-out sensitivity analyses showed stable results (Figure S6). Eleven studies (13 cohorts) reported an association between PFS and LIPI (0 vs. 1 + 2) [24, 26, 28, 29, 31, 35, 39, 42, 45, 49, 51], and the fixed effects model indicated that the pooled HR was 1.65 (95% CI: 1.46–1.86, *p* < 0.001, I^2^ = 10%, Figure S7).

Subgroup analyses were performed (0 vs. 1 and 0 vs. 2) on PFS based on retrospective or prospective design and tumour types. The findings demonstrated consistent outcomes across all subgroups, indicating the stability of the results (Table S2).

### Tumour response

The secondary point (ORR) was reported in 12 studies that included 3849 patients [18, 20–23, 29, 33, 35, 36, 44, 47, 50]. The pooled OR (0 vs. 1) was 0.63 (95% CI: 0.54–0.75, *p* < 0.001, I^2^ = 0%, Figure S8), suggesting that LIPI 1 was associated with worse tumor response. Similarly, the pooled OR (0 vs. 2) was 0.38 (95% CI: 0.29–0.50, *p* < 0.001, I^2^ = 31%, Figure S8), indicating that a higher LIPI score indeed suggested a worse tumour response.

Similarly, the secondary point (DCR) was reported in 9 studies that included 1632 patients [18, 20–23, 29, 35, 44, 47]. The pooled OR (0 vs. 1) was 0.47 (95% CI: 0.35–0.61, *p* < 0.001, I^2^ = 42%, Figure S9) and the pooled OR (0 vs. 2) was 0.19 (95% CI: 0.12–0.30, *p* < 0.001, I^2^ = 38%, Figure S9). Similar results were obtained after conducting a sensitivity analysis (Figure S10).

### Immune-related adverse events (irAEs)

In addition, we investigated studies that reported the association between ancillary endpoint (irAEs) and LIPI. A total of 2 studies reported by Pierro et al. [23] and Sonehara et al. [48] both showed that low points of LIPI were associated with a high occurrence of irAEs which were fully reported to be related to better clinical outcomes. Pierro et al. reported that they observed a greater rate of irAEs in the good LIPI group, with 17 events (45%) vs. 26 in the intermediate LIPI group (31%) and 13 (30%) in the poor LIPI group. Similarly, Sonehara et al. reported that the development of irAEs was independently predicted by a LIPI score of 0 or 1 (ORR: 0.200, 95% CI: 0.088–0.693, *p* = 0.011).

### Publication bias

Publication bias was evaluated through funnel plots, the Egger’s test, and the Begg’s test. The funnel plots exhibited approximate symmetry (Figure 1S11A-H and Figure S12 A-B), while the results of the Egger’s test and Begg’s test indicated the presence of publication bias in the studies examining the relationship between LIPI 1 or 2 and OS (Table 2). The trim-and-fill method identified a need to add six (OS: 0 vs. 1) or five (OS: 0 vs. 2) potential unpublished studies (Figure S12 C-D), and this did not significantly alter the outcome, which yielded pooled HRs of 1.79 (95% CI: 1.64–1.94, *p* < 0.001; OS: 0 vs. 1) and 3.48 (95% CI: 2.81–4.31, *p* < 0.001; OS: 0 vs. 2).

**Table 2:** Publication bias

Description	P value of Egger test	P value of Begg test	Corresponding funnel plot	Corresponding forest plot
OS: 0 vs. 1	0.0131	0.0006	Figure S11A	Figure 3
OS: 0 vs. 2	0.0846	0.0230	Figure S11B	Figure 3
OS: 0 vs. 1 + 2	0.2032	0.7290	Figure S12A	Figure S3
PFS: 0 vs. 1	0.6978	0.5285	Figure S11C	Figure 4
PFS: 0 vs. 2	0.6797	0.9343	Figure S11D	Figure 4
PFS: 0 vs. 1 + 2	0.4826	0.2988	Figure S12B	Figure S7
ORR: 0 vs. 1	0.1949	0.8909	Figure S11E	Figure S8
ORR: 0 vs. 2	0.4115	0.7839	Figure S11F	Figure S8
DCR: 0 vs. 1	0.7624	0.5316	Figure S11G	Figure S9
DCR: 0 vs. 2	0.5354	0.8348	Figure S11H	Figure S9

Note. OS: overall survival; PFS: progression-free survival; ORR: objective response rate; DCR: disease control rate

## Discussion

There is a growing body of evidence regarding the prognostic importance of peripheral blood inflammatory indices in various tumour types and settings [54–56]. In contrast to measuring biomarkers such as PD-L1, TMB, and MSI, taking routine blood samples offers greater accessibility and does not entail supplementary expenses, making the associated biomarkers readily applicable in real-world scenarios [5]. Therefore, many blood-based biomarkers have been developed for predicting the efficacy of cancer immunotherapy or monitoring the progression of tumours [57–59].

Among these biomarkers, the neutrophil-to-lymphocyte ratio (NLR), which reflects the systemic immune response to cancer-related inflammation, a hallmark of the initiation and progression of malignant types of cancer, has been the most studied. From a biological perspective, the NLR serves as an indicator of systemic inflammation and may provide insights into the immune system’s equilibrium in the presence of malignancy [54]. The neutrophil count is believed to mirror the inflammatory microenvironment, which in turn facilitates tumour-promoting processes such as cancer cell proliferation, metastasis, angiogenesis, and evasion of adaptive immune responses [60]. Conversely, lymphocytes possess potent abilities to suppress cancer progression, and their presence, particularly within the tumor microenvironment, is considered indicative of host immunity [61]. The elevation of LDH levels can be attributed to the heightened glycolytic activity of the tumour and tumour necrosis caused by hypoxia, with the latter being correlated with a substantial tumour burden [62]. Both glycolysis and hypoxia play a role in fostering an immunosuppressive microenvironment and impair the efficacy of immunotherapy [63]. Notably, Mezquita et al. reported that the LIPI score was an immunotherapy-specific prognostic factor and the LIPI could not stratify the prognosis of the chemotherapy cohort [18]. Kazandjian et al. then pooled the results of eleven randomized trials and found that LIPI was also a good prognostic predictor in patients with metastatic NSCLC undergoing chemotherapy [17]. Subsequently, a considerable number of studies have investigated the prognostic effect of LIPI in nonimmunotherapy patients. For example, LIPI can be used as a prognostic factor in patients with NSCLC or pancreatic cancer receiving radiotherapy, surgery, or tyrosine kinase inhibitors [64, 65]. Considering that LIPI can play a prognostic role in a variety of tumours and treatment modalities, LIPI may function as a universal prognostic predictor for cancer patients [66].

Thus far, this study included 35 studies (40 cohorts), and 9959 patients represented the largest meta-analysis comprehensively summarizing the prognostic value of LIPI in cancer patients treated with ICIs. Although one previous meta-analysis reported the prognostic value of LIPI in cancer patients treated with ICIs [67], it included only 12 studies and 4883 patients. Therefore, they only confirmed that the prognosis was significantly worse in the poor or intermediate LIPI group than in the good LIPI group. The difference in the ability of prognosis stratification between LIPI 1 and LIPI 2 patients remains unclear. In addition, they included studies published in conference abstracts with limited information resulting in limited assessment. A more extensive literature search was performed in this study and we included more cancer patients, accessing more data regarding various tumours and building a good basis for evaluating the prognostic value of LIPI in a pancancer setting. Moreover, Baujat plots and sensitivity analyses were conducted to ascertain the origins of heterogeneity and validate the stability of the obtained outcomes.

Our results represent three findings. First, intermediate or poor LIPI was significantly associated with poor OS, shorter PFS, and worse tumour response in cancer patients treated with ICIs. Specifically, compared to patients with LIPI 0, patients with LIPI 1 had a 1.69-fold greater risk of death, a 1.41-fold greater risk of progression, and a 0.63-fold lower odds of tumour response; similarly, compared to patients with LIPI 0, patients with LIPI 2 had a 3.03-fold greater risk of death, a 2.23-fold greater risk of progression, and a 0.38-fold lower odds of tumour response. Second, the LIPI classification system could robustly stratify the long-term prognosis and short-term treatment efficacy into 3 groups among the cancer patients receiving ICIs. Finally, given that LIPI was developed from NSCLC cohorts, we validated the prognostic value of LIPI in NSCLC patients and non-NSCLC patients. The results indicated that the stratification ability of LIPI was similar in NSCLC patients and non-NSCLC patients.

However, this meta-analysis and systematic review had several limitations. First, some of the included studies were retrospective studies, which led to inevitable selection bias and confounding bias. However, the subgroup analysis of the prospective studies (10 studies and 5110 patients) provided similar results to the overall results or the results of retrospective studies (27 studies and 4849 patients). Second, high heterogeneity was observed in some of the results, but Baujat plots were used to determine the source of heterogeneity. In addition, sensitivity analyses and subgroup analyses validated the stability of the results. Finally, publication bias was observed in some results, however, similar results were achieved with the Trim and Fill method. Despite these limitations, the results of the present study were reliable because low heterogeneity was detected and publication bias was not observed among most of the results. Moreover, with the rapid development of antitumour agents, it is necessary to explore the prognostic ability of LIPI in cancer patients receiving other types of immunotherapies (vaccine, adoptive cell transfer, and cytokine therapy) or antibody-drug conjugates in future studies. In addition, comparative studies for markers of systemic inflammatory responses, including LIPI, Glasgow score, NLR, lymphocyte monocyte ratio (LMR), platelet lymphocyte ratio (PLR), need to be carried out in the future to determine their prognostic role in different solid cancers.

## Conclusions

In patients with NSCLC or other solid tumours, the lung immune prognostic index could robustly stratify clinical outcomes into three groups among the patients receiving ICIs. LIPI is a low-cost, simple, accessible, and accurate prognostic tool in a pancancer setting and it may contribute to the evaluation of risk stratification in patients treated with ICIs.

### Electronic supplementary material

Below is the link to the electronic supplementary material.

Supplementary Material 1

## Data Availability

The datasets used and/or analysed during the current study are available from the corresponding author upon reasonable request.

## References

[1] Marin-Acevedo JA, Dholaria B, Soyano AE, Knutson KL, Chumsri S, Lou Y (2018). Next generation of immune checkpoint therapy in cancer: new developments and challenges. J Hematol Oncol.

[2] Yarchoan M, Johnson BA, Lutz ER, Laheru DA, Jaffee EM (2017). Targeting neoantigens to augment antitumour immunity. Nat Rev Cancer.

[3] Woroniecka K, Chongsathidkiet P, Rhodin K, Kemeny H, Dechant C, Farber SH (2018). T-Cell exhaustion signatures vary with tumor type and are severe in Glioblastoma. Clin cancer Research: Official J Am Association Cancer Res.

[4] Gu SS, Wang X, Hu X, Jiang P, Li Z, Traugh N (2020). Clonal tracing reveals diverse patterns of response to immune checkpoint blockade. Genome Biol.

[5] Guo Y, Xiang D, Wan J, Yang L, Zheng C. Focus on the dynamics of Neutrophil-to-lymphocyte ratio in Cancer patients treated with Immune Checkpoint inhibitors: a Meta-analysis and systematic review. Cancers (Basel). 2022;14(21). 10.3390/cancers14215297 PMC9658132 36358716

[6] Büttner R, Longshore JW, López-Ríos F, Merkelbach-Bruse S, Normanno N, Rouleau E (2019). Implementing TMB measurement in clinical practice: considerations on assay requirements. ESMO open.

[7] André T, Shiu KK, Kim TW, Jensen BV, Jensen LH, Punt C (2020). Pembrolizumab in microsatellite-instability-high Advanced Colorectal Cancer. N Engl J Med.

[8] Herbst RS, Giaccone G, de Marinis F, Reinmuth N, Vergnenegre A, Barrios CH (2020). Atezolizumab for First-Line treatment of PD-L1-Selected patients with NSCLC. N Engl J Med.

[9] Guven DC, Sahin TK, Erul E, Cakir IY, Ucgul E, Yildirim HC et al. The Association between Early Changes in Neutrophil-Lymphocyte Ratio and Survival in Patients Treated with Immunotherapy. J Clin Med. 2022;11(15). 10.3390/jcm11154523 PMC9369683 35956139

[10] Nakao S, Arai Y, Tasaki M, Yamashita M, Murakami R, Kawase T et al. Intratumoral expression of IL-7 and IL-12 using an oncolytic virus increases systemic sensitivity to immune checkpoint blockade. Sci Transl Med. 2020;12(526). 10.1126/scitranslmed.aax7992 31941828

[11] Sionov RV, Fridlender ZG, Granot Z (2015). The multifaceted roles neutrophils play in the Tumor Microenvironment. Cancer Microenvironment: Official J Int Cancer Microenvironment Soc.

[12] Chen DS, Mellman I (2013). Oncology meets immunology: the cancer-immunity cycle. Immunity.

[13] Templeton AJ, McNamara MG, Šeruga B, Vera-Badillo FE, Aneja P, Ocaña A (2014). Prognostic role of neutrophil-to-lymphocyte ratio in solid tumors: a systematic review and meta-analysis. J Natl Cancer Inst.

[14] Weide B, Martens A, Hassel JC, Berking C, Postow MA, Bisschop K (2016). Baseline Biomarkers for Outcome of Melanoma Patients Treated with Pembrolizumab. Clin cancer Research: Official J Am Association Cancer Res.

[15] Sakae H, Kanzaki H, Nasu J, Akimoto Y, Matsueda K, Yoshioka M (2017). The characteristics and outcomes of small bowel adenocarcinoma: a multicentre retrospective observational study. Br J Cancer.

[16] Petrelli F, Cabiddu M, Coinu A, Borgonovo K, Ghilardi M, Lonati V (2015). Prognostic role of lactate dehydrogenase in solid tumors: a systematic review and meta-analysis of 76 studies. Acta Oncol (Stockholm Sweden).

[17] Kazandjian D, Gong Y, Keegan P, Pazdur R, Blumenthal GM (2019). Prognostic value of the lung Immune Prognostic Index for patients treated for metastatic non-small cell Lung Cancer. JAMA Oncol.

[18] Mezquita L, Auclin E, Ferrara R, Charrier M, Remon J, Planchard D (2018). Association of the lung Immune Prognostic Index with Immune checkpoint inhibitor outcomes in patients with Advanced Non-small Cell Lung Cancer. JAMA Oncol.

[19] Xie J, Chen M, Han H, Xu K, Qiu G, Lin X (2023). Clinical impact of first-line PD-1 or PD-L1 inhibitors combined with chemotherapy in extensive-stage small cell lung cancer patients: a real-world multicenter propensity score-matched study. Thorac cancer.

[20] Sun T, Guo Y, Sun B, Chen L, Ren Y, Zhu L (2023). Association of the pretreatment lung immune prognostic index with immune checkpoint inhibitor outcomes in patients with advanced hepatocellular carcinoma. Eur J Med Res.

[21] Parent P, Auclin E, Patrikidou A, Mezquita L, Martínez Chanzá N, Dumont C et al. Prognostic value of the lung Immune Prognosis Index score for patients treated with Immune Checkpoint inhibitors for Advanced or metastatic urinary tract carcinoma. Cancers (Basel). 2023;15(4). 10.3390/cancers15041066 PMC9954148 36831409

[22] García-Corbacho J, Indacochea A, González Navarro AE, Victoria I, Moreno D, Pesántez D (2023). Determinants of activity and efficacy of anti-PD1/PD-L1 therapy in patients with advanced solid tumors recruited in a clinical trials unit: a longitudinal prospective biomarker-based study. Cancer Immunol Immunotherapy: CII.

[23] Pierro M, Baldini C, Auclin E, Vincent H, Varga A, Martin Romano P et al. Predicting Immunotherapy outcomes in older patients with solid tumors using the LIPI score. Cancers (Basel). 2022;14(20). 10.3390/cancers14205078 PMC9600023 36291861

[24] Pan Y, Si H, Jia R, Deng G, Yan H, Fan M (2022). Analysis of a systemic inflammatory biomarker in Advanced Bile Tract Carcinoma Treated with Anti-PD-1 therapy: prognostic and predictive significance of Lung Immune Prognostic Index score. J Oncol.

[25] Gou M, Qian N, Zhang Y, Wei L, Fan Q, Wang Z (2022). Construction of a nomogram to predict the survival of metastatic gastric cancer patients that received immunotherapy. Front Immunol.

[26] Chen S, Guo S, Gou M, Pan Y, Fan M, Zhang N (2022). A composite indicator of derived neutrophil-lymphocyte ratio and lactate dehydrogenase correlates with outcomes in pancreatic carcinoma patients treated with PD-1 inhibitors. Front Oncol.

[27] Assié JB, Crépin F, Grolleau E, Canellas A, Geier M, Grébert-Manuardi A et al. Immune-Checkpoint inhibitors for malignant pleural mesothelioma: a French, Multicenter, Retrospective Real-World Study. Cancers (Basel). 2022;14(6). 10.3390/cancers14061498 PMC8946798 35326648

[28] Li L, Pi C, Yan X, Lu J, Yang X, Wang C (2021). Prognostic value of the pretreatment lung Immune Prognostic Index in Advanced Small Cell Lung Cancer patients treated with First-Line PD-1/PD-L1 inhibitors plus chemotherapy. Front Oncol.

[29] Hou B, Wang P, Liu T, Chen S, Li T, Zhang S (2021). Association of the pretreatment lung immune prognostic index with survival outcomes in advanced gastric cancer patients treated with immune checkpoint inhibitors. Clin Res Hepatol Gastroenterol.

[30] Auclin E, Vuagnat P, Smolenschi C, Taieb J, Adeva J, Nebot-Bral L (2021). Association of the lung Immune Prognostic Index with Immunotherapy outcomes in Mismatch Repair deficient tumors. Cancers (Basel).

[31] Chen S, Huang Z, Jia W, Tao H, Zhang S, Ma J (2020). Association of the pretreatment lung Immune Prognostic Index with Survival outcomes in Advanced Hepatocellular Carcinoma patients treated with PD-1 inhibitors. J Hepatocellular Carcinoma.

[32] Al Darazi G, Martin E, Delord JP, Korakis I, Betrian S, Estrabaut M et al. Improving patient selection for immuno-oncology phase 1 trials: external validation of six prognostic scores in a French Cancer Center. Int J Cancer. 2020. 10.1002/ijc.33409 33231298

[33] Meyers DE, Stukalin I, Vallerand IA, Lewinson RT, Suo A, Dean M et al. The Lung Immune Prognostic Index discriminates survival outcomes in patients with Solid Tumors Treated with Immune Checkpoint inhibitors. Cancers (Basel). 2019;11(11). 10.3390/cancers11111713 PMC6896022 31684111

[34] Moher D, Liberati A, Tetzlaff J, Altman DG (2009). Preferred reporting items for systematic reviews and meta-analyses: the PRISMA statement. PLoS Med.

[35] Ruiz-Bañobre J, Areses-Manrique MC, Mosquera-Martínez J, Cortegoso A, Afonso-Afonso FJ, de Dios-Álvarez N (2019). Evaluation of the lung immune prognostic index in advanced non-small cell lung cancer patients under nivolumab monotherapy. Translational lung cancer Res.

[36] Sorich MJ, Rowland A, Karapetis CS, Hopkins AM (2019). Evaluation of the lung Immune Prognostic Index for Prediction of Survival and response in patients treated with atezolizumab for NSCLC: pooled analysis of clinical trials. J Thorac Oncology: Official Publication Int Association Study Lung Cancer.

[37] Mazzaschi G, Minari R, Zecca A, Cavazzoni A, Ferri V, Mori C (2020). Soluble PD-L1 and circulating CD8 + PD-1 + and NK cells enclose a Prognostic and Predictive Immune Effector score in Immunotherapy treated NSCLC patients. Lung cancer (Amsterdam. Netherlands).

[38] Wang W, Huang Z, Yu Z, Zhuang W, Zheng W, Cai Z (2020). Prognostic value of the lung Immune Prognostic Index May Differ in patients treated with Immune checkpoint inhibitor monotherapy or combined with chemotherapy for non-small cell Lung Cancer. Front Oncol.

[39] Ali WAS, Hui P, Ma Y, Wu Y, Zhang Y, Chen Y (2021). Determinants of survival in advanced non-small cell lung cancer patients treated with anti-PD-1/PD-L1 therapy. Ann Transl Med.

[40] Grosjean HAI, Dolter S, Meyers DE, Ding PQ, Stukalin I, Goutam S et al. Effectiveness and safety of First-Line Pembrolizumab in older adults with PD-L1 positive non-small cell Lung Cancer: a retrospective cohort study of the Alberta Immunotherapy Database. Current oncology (Toronto, Ont). 2021;28(5):4213–22. 10.3390/curroncol28050357 PMC8534423 34677275

[41] Hopkins AM, Kichenadasse G, Abuhelwa AY, McKinnon RA, Rowland A, Sorich MJ. Value of the lung Immune Prognostic Index in patients with Non-small Cell Lung Cancer Initiating First-Line Atezolizumab Combination Therapy: Subgroup Analysis of the IMPOWER150 trial. Cancers (Basel). 2021;13(5). 10.3390/cancers13051176 PMC7967121 33803256

[42] Mountzios G, Samantas E, Senghas K, Zervas E, Krisam J, Samitas K (2021). Association of the advanced lung cancer inflammation index (ALI) with immune checkpoint inhibitor efficacy in patients with advanced non-small-cell lung cancer. ESMO open.

[43] Sheng J, Li H, Yu X, Yu S, Chen K, Pan G (2021). Efficacy of PD-1/PD-L1 inhibitors in patients with non-small cell lung cancer and brain metastases: a real-world retrospective study in China. Thorac cancer.

[44] Veccia A, Sforza V, Vattemi E, Inno A, Kinspergher S, Dipasquale M (2021). Pretreatment lung immune prognostic index as a biomarker in advanced non-small-cell lung cancer patients receiving first line pembrolizumab. Immunotherapy.

[45] Chen J, Wei S, Zhao T, Zhang X, Wang Y, Zhang X (2022). Clinical significance of serum biomarkers in Stage IV Non-small-cell Lung Cancer treated with PD-1 inhibitors: LIPI score, NLR, dNLR, LMR, and PAB. Dis Markers.

[46] De Giglio A, Tassinari E, Zappi A, Di Federico A, Lenzi B, Sperandi F et al. The Palliative Prognostic (PaP) score without clinical evaluation predicts early mortality among Advanced NSCLC patients treated with immunotherapy. Cancers (Basel). 2022;14(23). 10.3390/cancers14235845 PMC9739118 36497326

[47] Ortega-Franco A, Hodgson C, Raja H, Carter M, Lindsay C, Hughes S (2022). Real-World Data on Pembrolizumab for pretreated non-small-cell Lung Cancer: clinical outcome and relevance of the lung Immune Prognostic Index. Target Oncol.

[48] Sonehara K, Tateishi K, Araki T, Komatsu M, Akahane J, Yamamoto H (2022). Predictive factors correlated with the development of Immune-related adverse events in patients with Non-small Cell Lung Cancer treated with Immune Checkpoint inhibitors. Cancer Manage Res.

[49] Uehara Y, Hakozaki T, Kitadai R, Narita K, Watanabe K, Hashimoto K (2022). Association between the baseline tumor size and outcomes of patients with non-small cell lung cancer treated with first-line immune checkpoint inhibitor monotherapy or in combination with chemotherapy. Translational lung cancer Res.

[50] Diker O, Olgun P, Balyemez U, Sigit Ikiz S (2023). Development of a Novel Predictive-Prognostic Scoring Index for Immune checkpoint inhibitors in Advanced Non-small Cell Lung Cancer. Cureus.

[51] Inoue Y, Inui N, Karayama M, Asada K, Matsuura S, Ikeda M (2023). Serum immune modulators associated with immune-related toxicities and efficacy of atezolizumab in patients with non-small cell lung cancer. J Cancer Res Clin Oncol.

[52] Knetki-Wróblewska M, Tabor S, Piórek A, Płużański A, Winiarczyk K, Zaborowska-Szmit M et al. Nivolumab or Atezolizumab in the second-line treatment of Advanced Non-small Cell Lung Cancer? A Prognostic Index based on data from Daily Practice. J Clin Med. 2023;12(6). 10.3390/jcm12062409 PMC10053214 36983409

[53] Hopkins AM, Wagner J, Kichenadasse G, Modi N, Rowland A, Sorich MJ (2020). Patient-reported outcomes as a prognostic marker of survival in patients with advanced nonsmall cell lung cancer treated with immunotherapy. Int J Cancer.

[54] Lalani AA, Xie W, Martini DJ, Steinharter JA, Norton CK, Krajewski KM (2018). Change in Neutrophil-to-lymphocyte ratio (NLR) in response to immune checkpoint blockade for metastatic renal cell carcinoma. J Immunother Cancer.

[55] Lu H, Yu C, Maimaiti M, Li G (2023). The predictive value of perioperative circulating markers on surgical complications in patients undergoing robotic-assisted radical prostatectomy. World J Surg Oncol.

[56] Iwasa YI, Shimizu M, Matsuura K, Hori K, Hiramatsu K, Sugiyama K (2023). Prognostic significance of pre- and post-treatment hematological biomarkers in patients with head and neck cancer treated with chemoradiotherapy. Sci Rep.

[57] Tan Q, Liu S, Liang C, Han X, Shi Y (2018). Pretreatment hematological markers predict clinical outcome in cancer patients receiving immune checkpoint inhibitors: a meta-analysis. Thorac cancer.

[58] Liu N, Mao J, Tao P, Chi H, Jia W, Dong C (2022). The relationship between NLR/PLR/LMR levels and survival prognosis in patients with non-small cell lung carcinoma treated with immune checkpoint inhibitors. Medicine.

[59] Takenaka Y, Oya R, Takemoto N, Inohara H (2022). Neutrophil-to-lymphocyte ratio as a prognostic marker for head and neck squamous cell carcinoma treated with immune checkpoint inhibitors: Meta-analysis. Head Neck.

[60] Mantovani A, Allavena P, Sica A, Balkwill F (2008). Cancer-related inflammation. Nature.

[61] Gooden MJ, de Bock GH, Leffers N, Daemen T, Nijman HW (2011). The prognostic influence of tumour-infiltrating lymphocytes in cancer: a systematic review with meta-analysis. Br J Cancer.

[62] Van Wilpe S, Koornstra R, Den Brok M, De Groot JW, Blank C, De Vries J (2020). Lactate dehydrogenase: a marker of diminished antitumor immunity. Oncoimmunology.

[63] Miholjcic TBS, Halse H, Bonvalet M, Bigorgne A, Rouanne M, Dercle L et al. Rationale for LDH-targeted cancer immunotherapy. European journal of cancer (Oxford, England: 1990). 2023;181:166– 78. 10.1016/j.ejca.2022.11.032 36657325

[64] Minami S, Ihara S, Komuta K (2019). Pretreatment lung Immune Prognostic Index is a prognostic marker of chemotherapy and epidermal growth factor receptor tyrosine kinase inhibitor. World J Oncol.

[65] Zhang T, Xue W, Wang D, Xu K, Wu L, Wu Y (2021). A validation study on the lung immune prognostic index for prognostic value in patients with locally advanced non-small cell lung cancer. Radiotherapy Oncology: J Eur Soc Therapeutic Radiol Oncol.

[66] Zhou Q, Deng G, Wang Z, Dai G (2022). Preoperative lung immune prognostic index predicts survival in patients with pancreatic cancer undergoing radical resection. Front Surg.

[67] Liu H, Yang XL, Yang XY, Dong ZR, Chen ZQ, Hong JG (2021). The prediction potential of the pretreatment lung Immune Prognostic Index for the therapeutic outcomes of Immune checkpoint inhibitors in patients with Solid Cancer: a systematic review and Meta-analysis. Front Oncol.

