# Supplementary material for: Prognosis stratification of cancer patients treated with immune checkpoint inhibitors through lung immune prognostic index: a meta-analysis and systematic review
Source: BMC Cancer. 2024 Apr 25;24:523. doi: 10.1186/s12885-024-12271-0 (PMC11047037; doi:10.1186/s12885-024-12271-0)
Supplement: Supplementary file 1 — Supplementary Material 1 [file 12885_2024_12271_MOESM1_ESM.docx]

**Supplementary Materials of Figures and Tables**

**Supplementary Materials Figures**

Figure S1 The Baujat plot (OS:0 vs 2)

Figure S2 Leave-one-out sensitivity analyses (OS:0 vs 2)

Figure S3 The forest plot (OS:0 vs 1+2)

Figure S4 The Baujat plot (OS:0 vs 1+2)

Figure S5 Leave-one-out sensitivity analyses (OS:0 vs 1+2)

Figure S6 Leave-one-out sensitivity analyses (PFS:0 vs 2)

Figure S7 The forest plot (OS:0 vs 1+2)

Figure S8 The forest plot (ORR:0 vs 1 and 0 vs 2)

Figure S9 The forest plot (DCR:0 vs 1 and 0 vs 2)

Figure S10 Leave-one-out sensitivity analyses (DCR:0 vs 2)

Figure S11 Funnel plots

Figure S12 Additional funnel plots

**Supplementary Materials Tables**

Table S1 Subgroup analyses of OS

Table S2 Subgroup analyses of PFS

**Figure S1 The Baujat plot (OS:0 vs 2)**


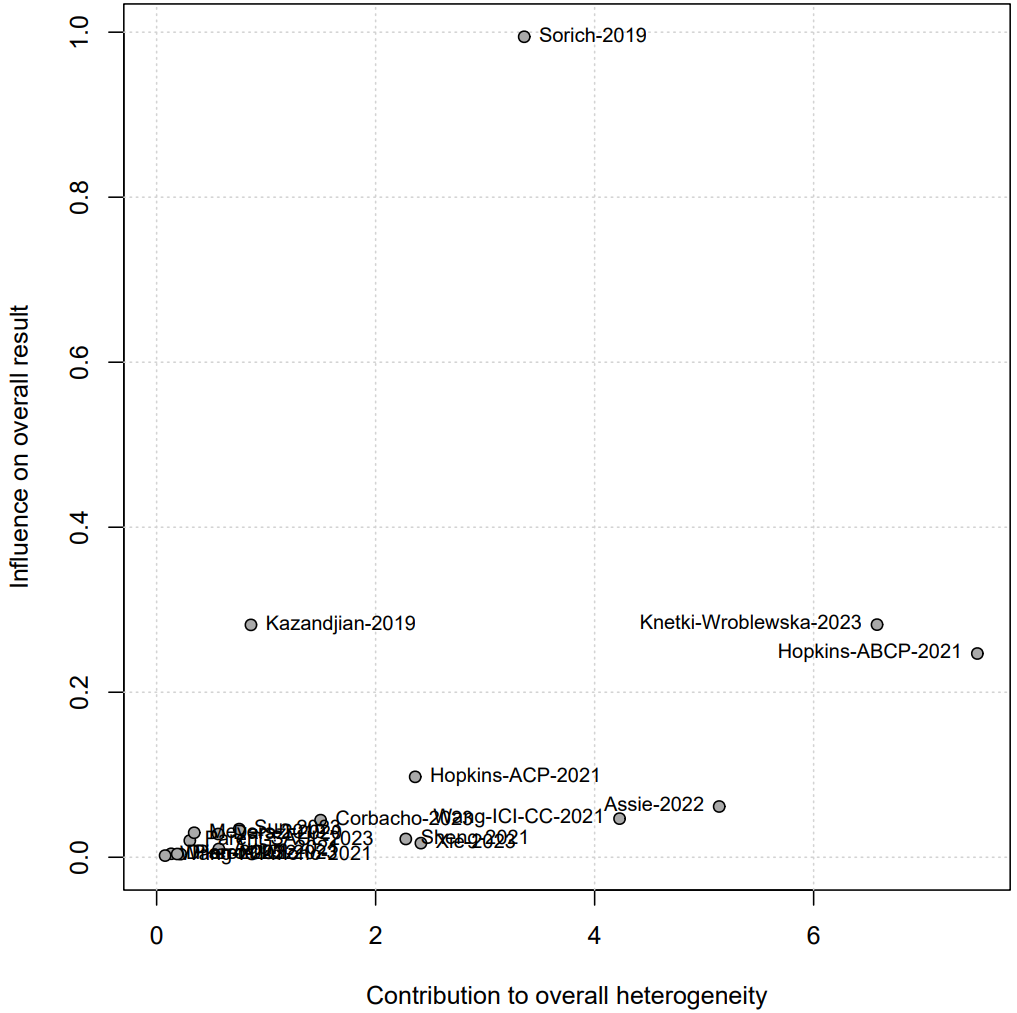


Baujat plots showed the potential source of heterogeneity when conducted the meta-analysis for OS and LIPI (0 vs 2)

**Figure S2 Leave-one-out sensitivity analyses (OS:0 vs 2)**


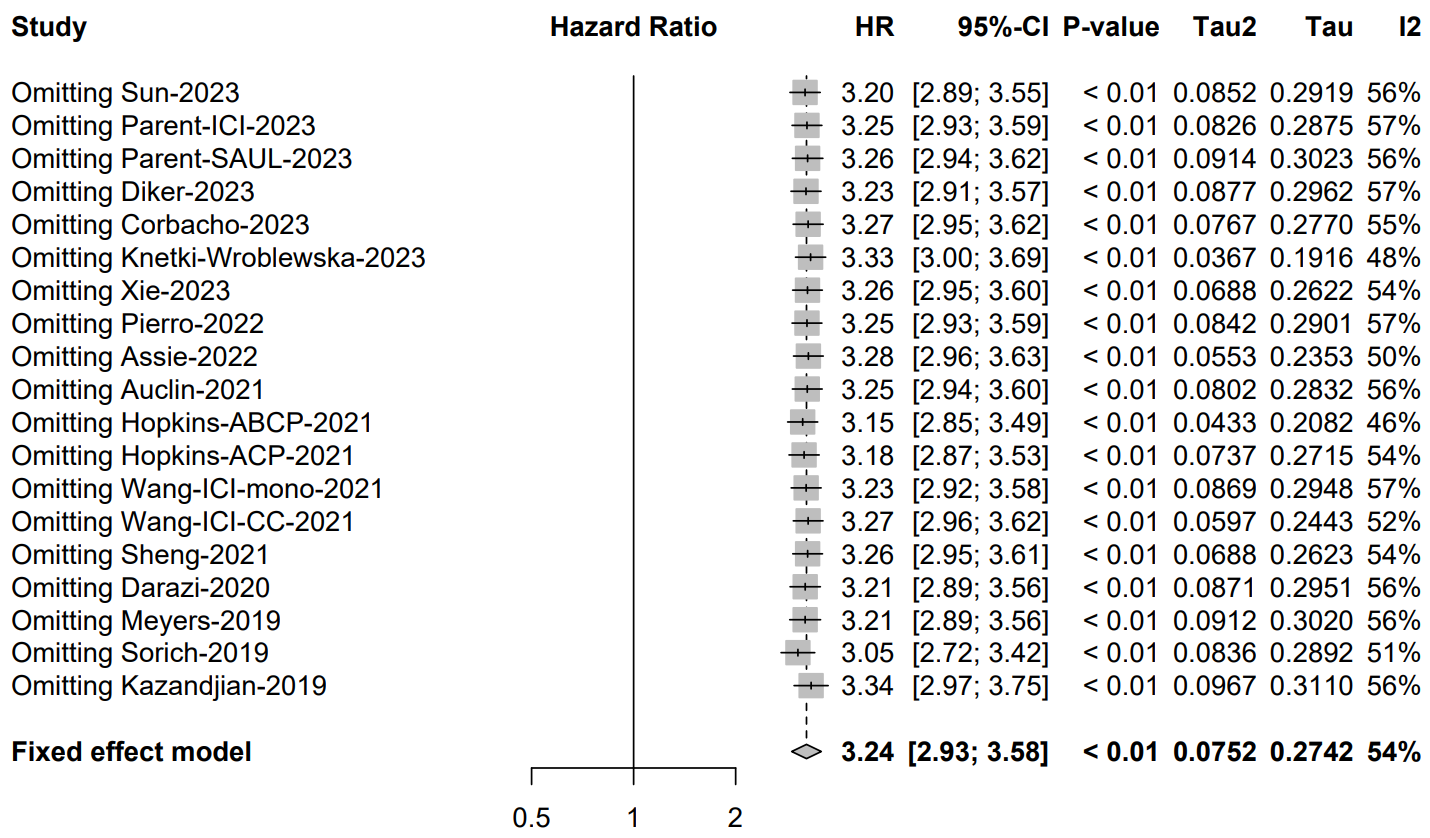


Leave-one-out sensitivity analyses assessed the results robustness of the meta-analysis for OS and LIPI (0 vs 2) by excluding the included studies one by one.

**Figure S3 The forest plot (OS:0 vs 1+2)**


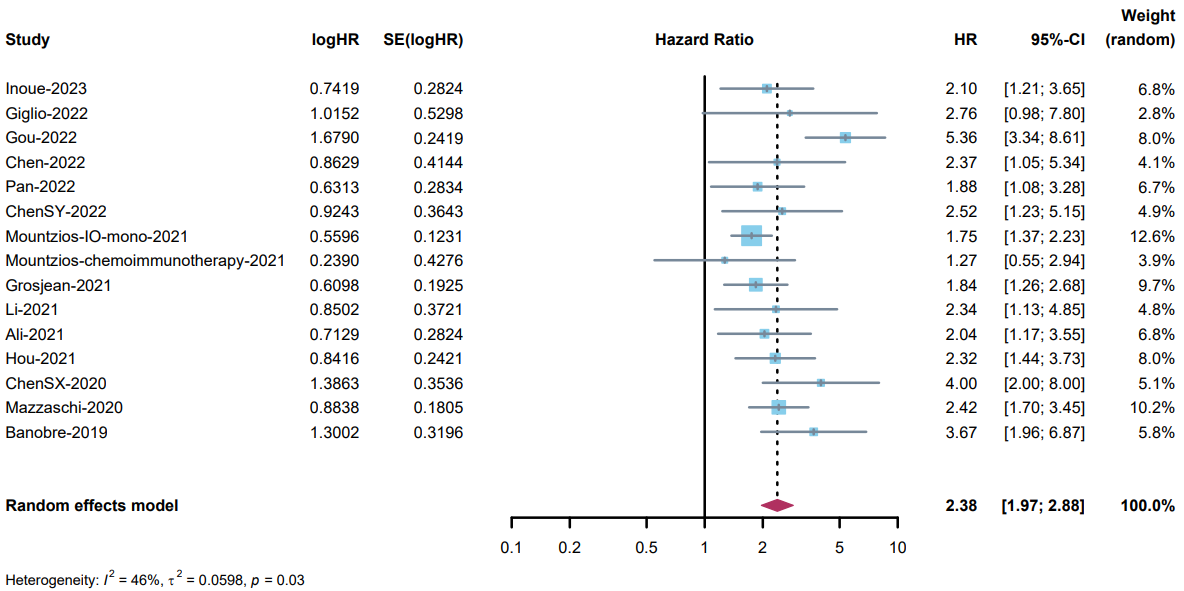


Forest plot showed the result of meta-analysis for OS and LIPI (0 vs 1+2) with a random effect model.

**Figure S4 The Baujat plot (OS:0 vs 1+2)**


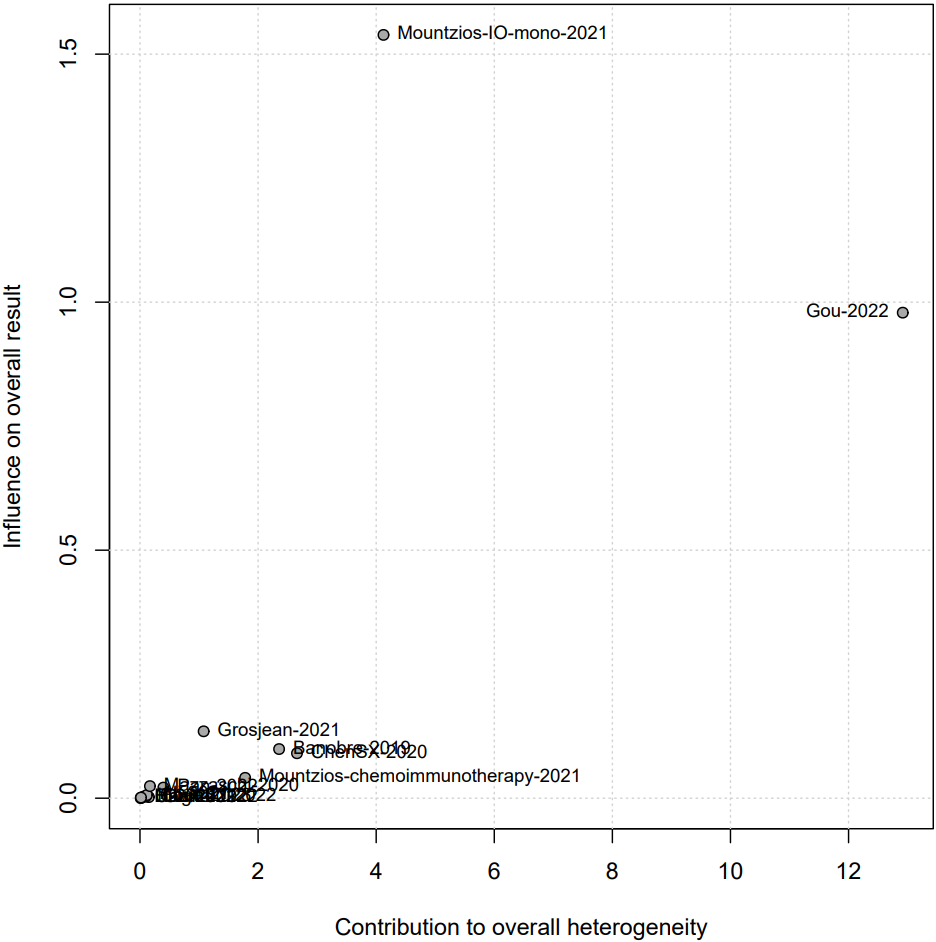


Baujat plots showed the potential source of heterogeneity when conducted the meta-analysis for OS and LIPI (0 vs 1+2)

**Figure S5 Leave-one-out sensitivity analyses (OS:0 vs 1+2)**


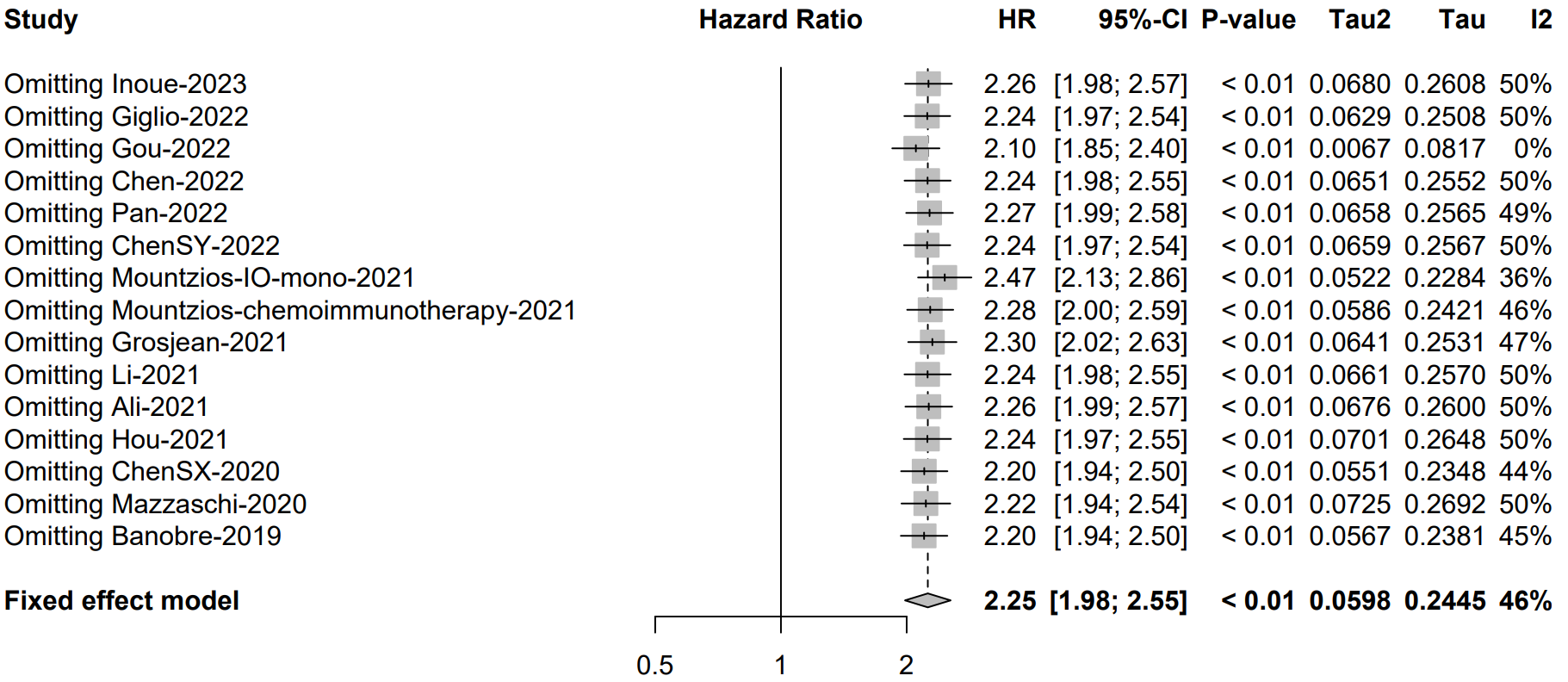


Leave-one-out sensitivity analyses assessed the results robustness of the meta-analysis for OS and LIPI (0 vs 1+2) by excluding the included studies one by one.

**Figure S6 Leave-one-out sensitivity analyses (PFS:0 vs 2)**


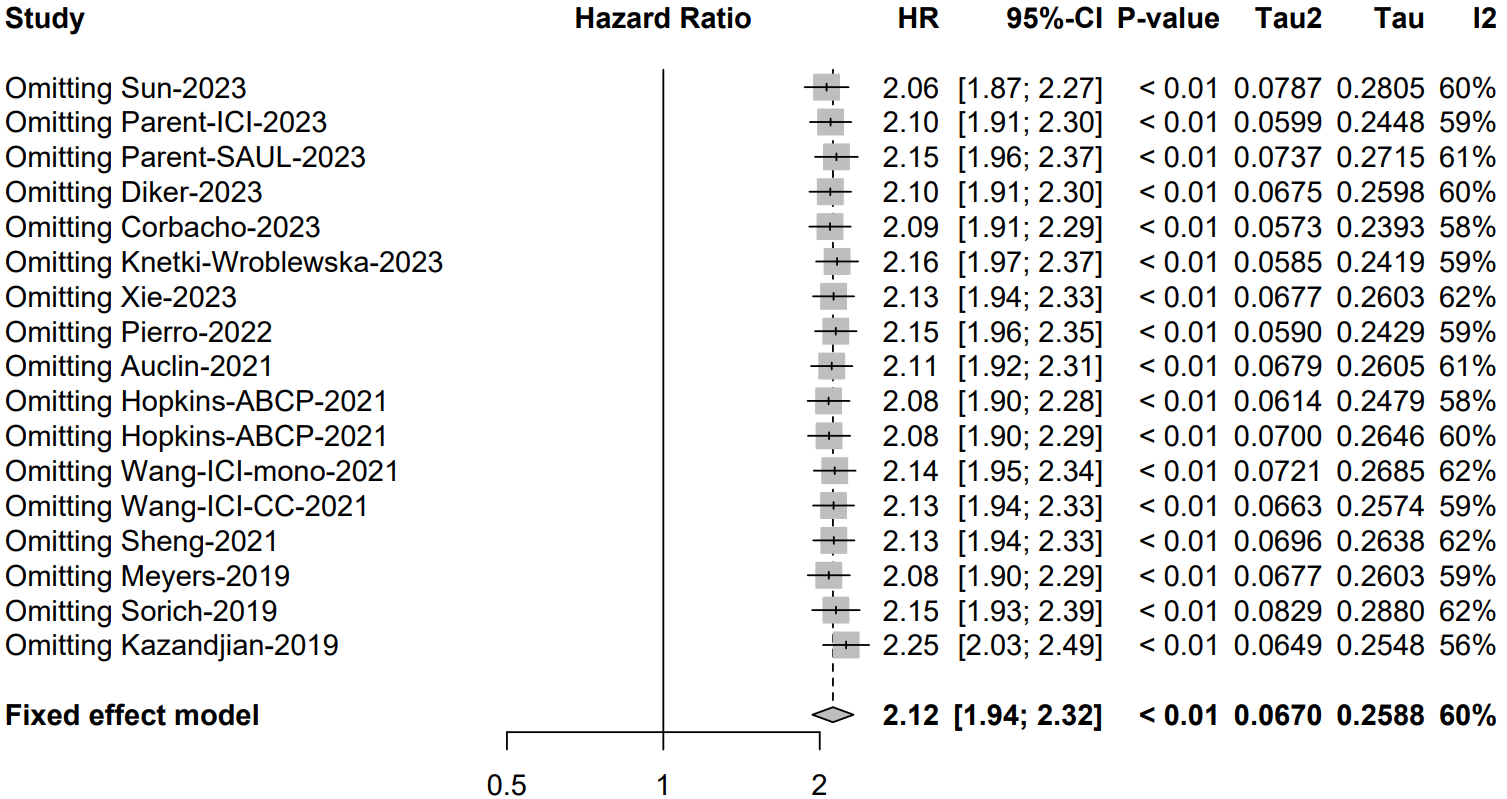


Leave-one-out sensitivity analyses assessed the results robustness of the meta-analysis for PFS and LIPI (0 vs 2) by excluding the included studies one by one.

**Figure S7 The forest plot (PFS:0 vs 1+2)**


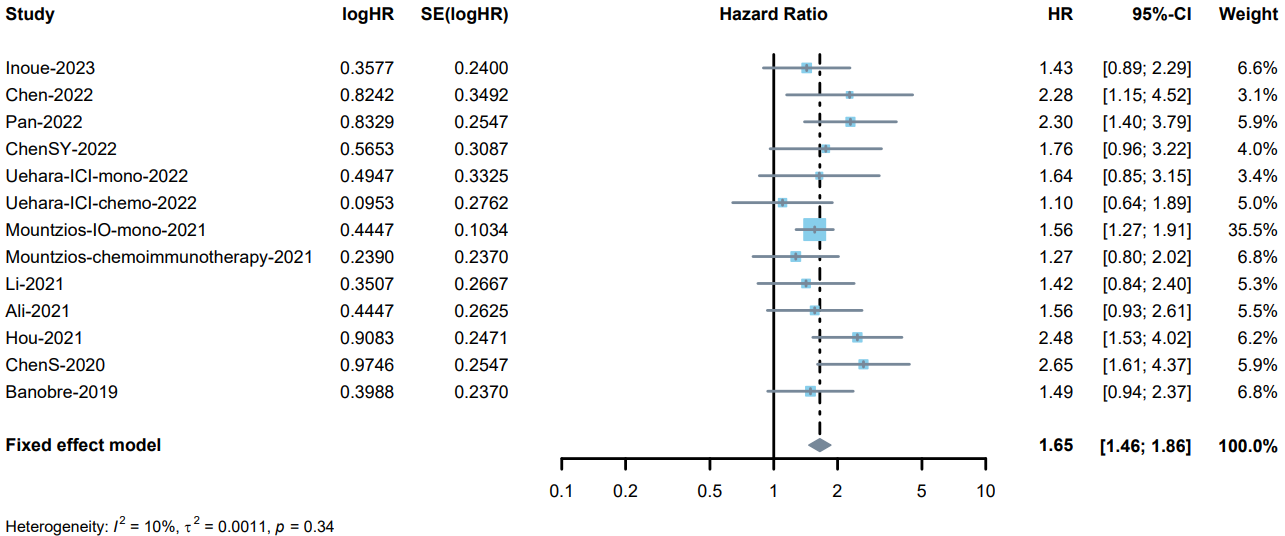


Forest plot showed the result of meta-analysis for PFS and LIPI (0 vs 1+2) with a fixed effect model.

**Figure S8 The forest plot (ORR:0 vs 1 and 0 vs 2)**


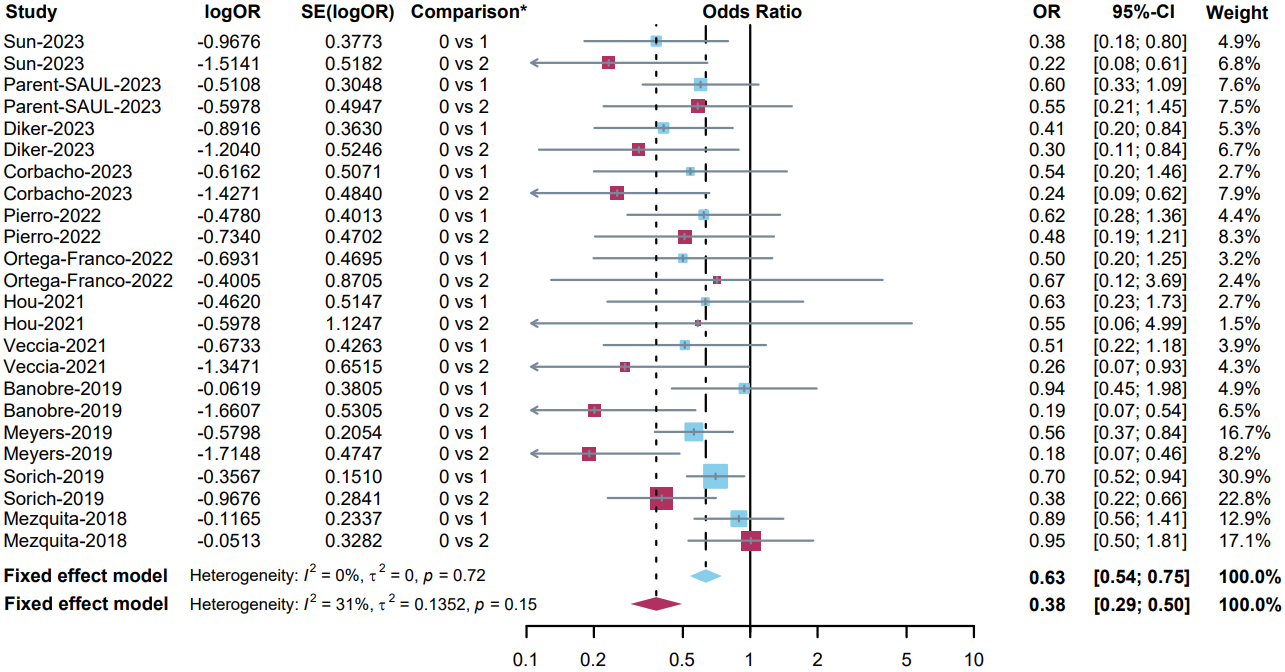


Forest plot showed the result of meta-analysis for ORR and LIPI (0 vs 1: blue; 0 vs 2: red) with fixed effect models.

**Figure S9 The forest plot (DCR:0 vs 1 and 0 vs 2)**


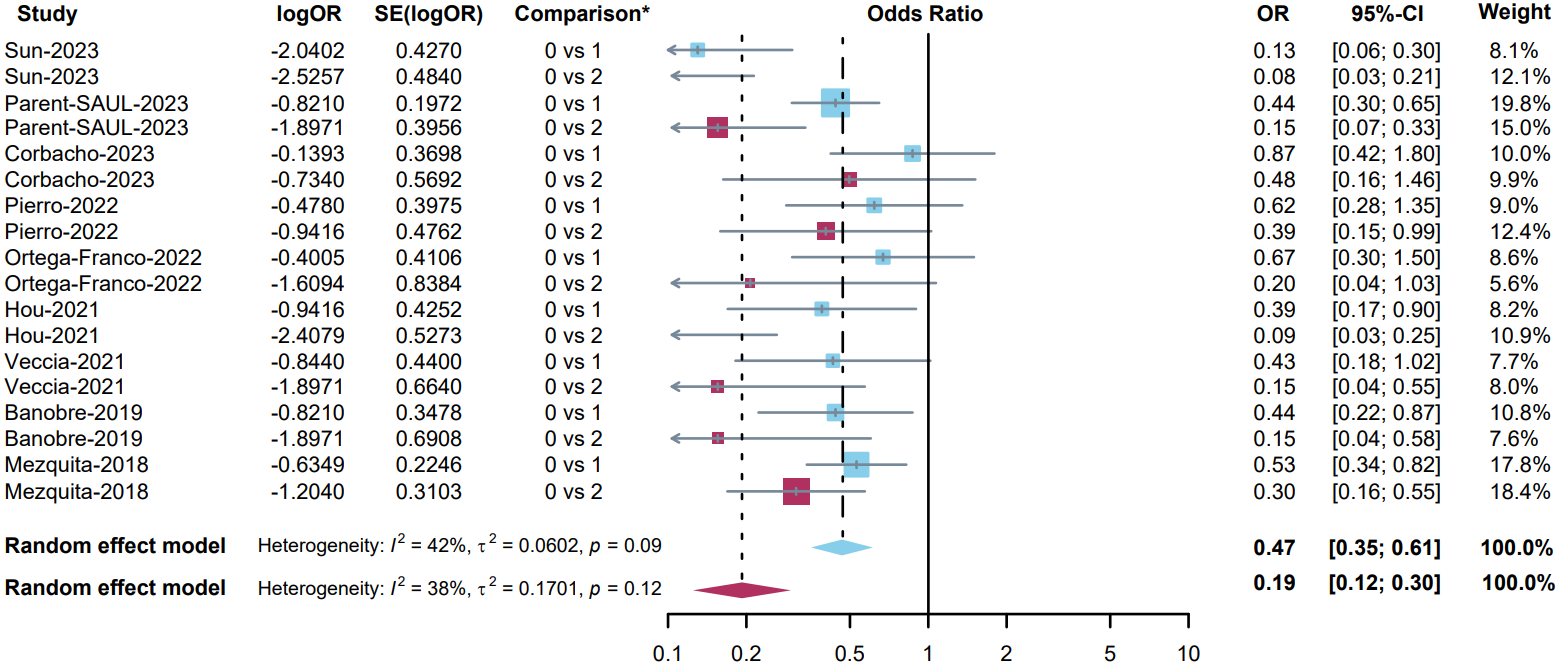


Forest plot showed the result of meta-analysis for DCR and LIPI (0 vs 1: blue; 0 vs 2: red) with random effect models.

**Figure S10 Leave-one-out sensitivity analyses (DCR:0 vs 2)**


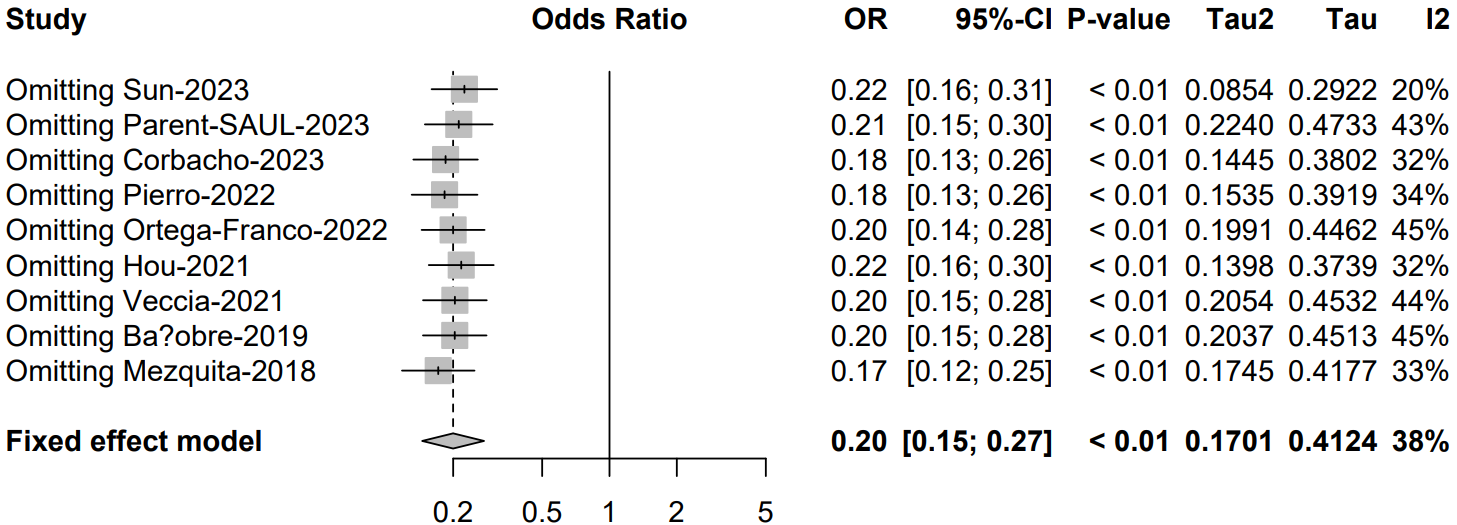


Leave-one-out sensitivity analyses assessed the results robustness of the meta-analysis for DCR and LIPI (0 vs 2) by excluding the included studies one by one.

**Figure S11 Funnel plots**


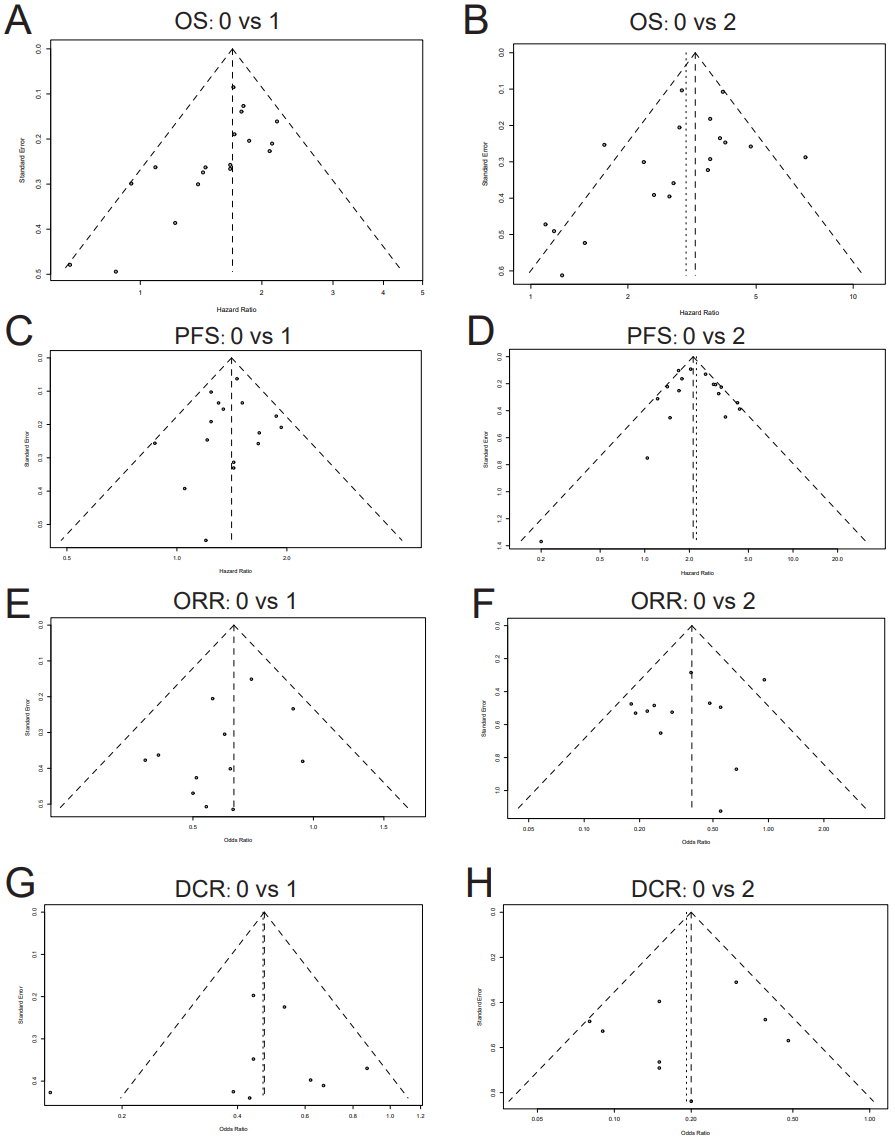


The funnel plots assessed the publication bias of the meta-analyses for OS (0 vs 1: A and 0 vs 2: B), PFS (0 vs 1: C and 0 vs 2: D), ORR (0 vs 1: E and 0 vs 2: F) and DCR (0 vs 1: G and 0 vs2: H)

**Figure S12 Additional funnel plots**


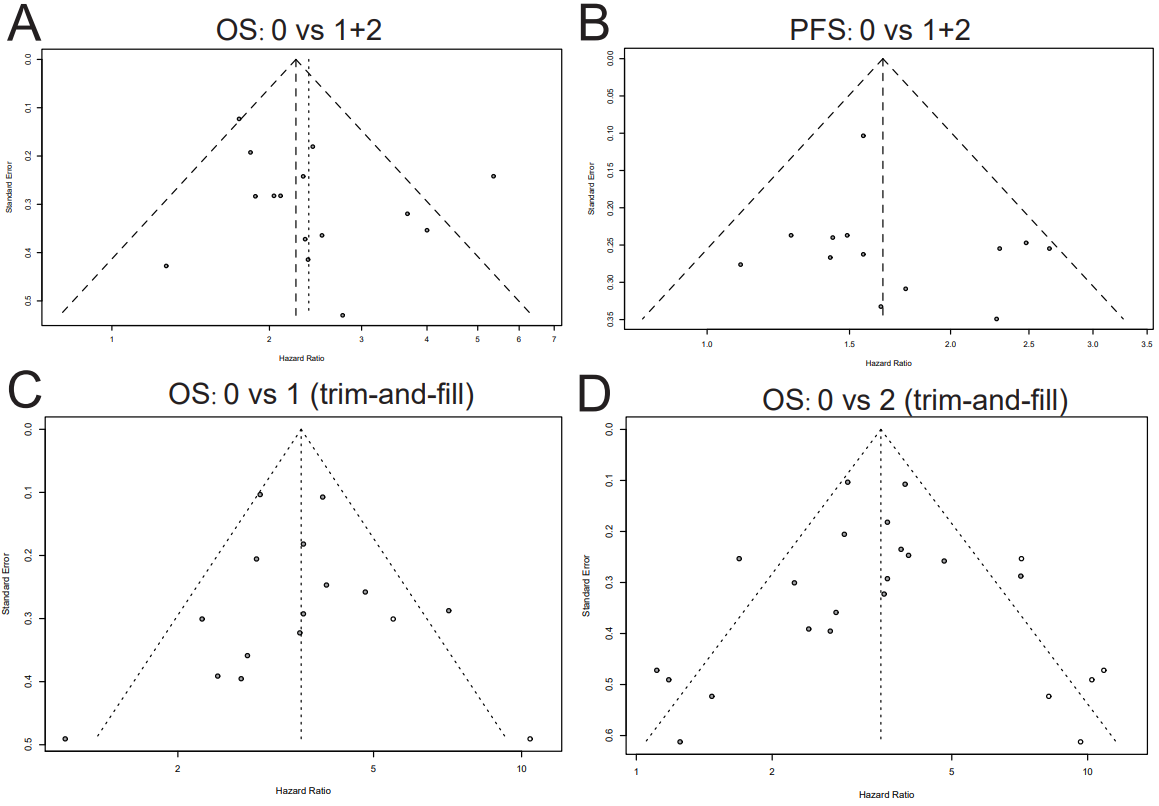


The funnel plots assessed the publication bias of the meta-analyses for OS (0 vs 1+2: A, 0 vs 1 [trim-and-fill]: C, 0 vs 2 [trim-and-fill]: D) and PFS (0 vs 1+2)

**Table S1 Subgroup analyses of overall survival**

| **Subgroup** | **No. of cohorts** | **No. of patients** | **Estimates**  **(HR)** | **Lower limit to Upper limit** | ***P*‐value** | **I*²*** | **Model** |
| --- | --- | --- | --- | --- | --- | --- | --- |
| **R or P**  **(0 vs 1)** |  |  |  |  |  |  |  |
| R | 13 | 2681 | 1.64 | 1.45-1.85 | <0.001 | 31% | Fixed |
| P | 5 | 2696 | 1.75 | 1.54-2.00 | <0.001 | 0% | Fixed |
| **Tumor types**  **(0 vs 1)** |  |  |  |  |  |  |  |
| NSCLC | 8 | 2925 | 1.71 | 1.51-1.94 | <0.001 | 0% | Fixed |
| Non-NSCLC | 10 | 2452 | 1.62 | 1.38-1.89 | <0.001 | 36% | Random |
| **R or P**  **(0 vs 2)** |  |  |  |  |  |  |  |
| R | 13 | 2774 | 2.60 | 2.08-3.25 | <0.001 | 39% | Random |
| P | 6 | 4064 | 3.79 | 2.92-4.91 | <0.001 | 64% | Random |
| **Tumor types**  **(0 vs 2)** |  |  |  |  |  |  |  |
| NSCLC | 9 | 4386 | 3.15 | 2.27-4.38 | <0.001 | 70% | Random |
| Non-NSCLC | 10 | 2452 | 3.03 | 2.55-3.60 | <0.001 | 21% | Fixed |

Note. R: Retrospective; P: Prospective; NSCLC: Non-Small Cell Lung Cancer.

**Table 2 Subgroup analyses of PFS**

| **Subgroup** | **No. of cohorts** | **No. of patients** | **Estimates**  **(HR)** | **Lower limit to Upper limit** | ***P*‐value** | **I*²*** | **Model** |
| --- | --- | --- | --- | --- | --- | --- | --- |
| **R or P**  **(0 vs 1)** |  |  |  |  |  |  |  |
| R | 12 | 2572 | 1.36 | 1.21-1.51 | <0.001 | 8% | Fixed |
| P | 4 | 2437 | 1.46 | 1.32-1.62 | <0.001 | 0% | Fixed |
| **Tumor types**  **(0 vs 1)** |  |  |  |  |  |  |  |
| NSCLC | 8 | 2925 | 1.44 | 1.31-1.58 | <0.001 | 0% | Fixed |
| Non-NSCLC | 8 | 2084 | 1.36 | 1.20-1.54 | <0.001 | 21% | Fixed |
| **R or P**  **(0 vs 2)** |  |  |  |  |  |  |  |
| R | 12 | 2665 | 2.10 | 1.66-2.66 | <0.001 | 55% | Random |
| P | 5 | 3805 | 2.47 | 1.82-3.36 | <0.001 | 74% | Random |
| **Tumor types**  **(0 vs 2)** |  |  |  |  |  |  |  |
| NSCLC | 9 | 4386 | 2.07 | 1.66-2.58 | <0.001 | 59% | Random |
| Non-NSCLC | 8 | 2084 | 2.48 | 1.83-3.35 | <0.001 | 58% | Random |

Note. R: Retrospective; P: Prospective; NSCLC: Non-Small Cell Lung Cancer.
